# Selective targeting of dipeptidyl‐peptidase 4 (DPP‐4) positive senescent chondrocyte ameliorates osteoarthritis progression

**DOI:** 10.1111/acel.14161

**Published:** 2024-03-31

**Authors:** Du Hyun Ro, Gun Hee Cho, Ji Yoon Kim, Seong Ki Min, Ha Ru Yang, Hee Jung Park, Sun Young Wang, You Jung Kim, Myung Chul Lee, Hyun Cheol Bae, Hyuk‐Soo Han

**Affiliations:** ^1^ Department of Orthopedic Surgery Seoul National University College of Medicine Seoul Korea; ^2^ Laboratory for Cellular Response to Oxidative Stress Cell2in, Inc. Seoul Korea; ^3^ Department of Orthopedic Surgery Seoul National University Hospital Seoul Korea

**Keywords:** dipeptidyl peptidase 4, osteoarthritis, senescence, senolytics, sitagliptin

## Abstract

Senescent cells increase in many tissues with age and induce age‐related pathologies, including osteoarthritis (OA). Senescent chondrocytes (SnCs) are found in OA cartilage, and the clearance of those chondrocytes prevents OA progression. However, targeting SnCs is challenging due to the absence of a senescent chondrocyte‐specific marker. Therefore, we used flow cytometry to screen and select senescent chondrocyte surface markers and cross‐validated with published transcriptomic data. Chondrocytes expressing dipeptidyl peptidase‐4 (DPP‐4), the selected senescent chondrocyte‐specific marker, had multiple senescence phenotypes, such as increased senescence‐associated‐galactosidase, p16, p21, and senescence‐associated secretory phenotype expression, and showed OA chondrocyte phenotypes. To examine the effects of DPP‐4 inhibition on DPP‐4+ SnCs, sitagliptin, a DPP‐4 inhibitor, was treated in vitro. As a result, DPP‐4 inhibition selectively eliminates DPP‐4+ SnCs without affecting DPP‐4‐ chondrocytes. To assess in vivo therapeutic efficacy of targeting DPP‐4+ SnCs, three known senolytics (ABT263, 17DMAG, and metformin) and sitagliptin were comparatively verified in a DMM‐induced rat OA model. Sitagliptin treatment specifically and effectively eliminated DPP‐4+ SnCs, compared to the other three senolytics. Furthermore, Intra‐articular sitagliptin injection to the rat OA model increased collagen type II and proteoglycan expression and physical functions and decreased cartilage destruction, subchondral bone plate thickness and MMP13 expression, leading to the amelioration of OA phenotypes. Collectively, OARSI score was lowest in the sitagliptin treatment group. Taken together, we verified DPP‐4 as a surface marker for SnCs and suggested that the selective targeting of DPP‐4+ chondrocytes could be a promising strategy to prevent OA progression.

## Graphical abstract

DPP‐4+ senescent chondrocytes, showing multiple senescence phenotypes such as decreased proliferation and anabolic factor expression and increased senescence marker and catabolic marker expression, accumulates in osteoarthritic cartilage. Sitagliptin treatment could selectively target and eliminate DPP‐4+ senescent chondrocytes, consequently leading to suppression of osteoarthritis progression.

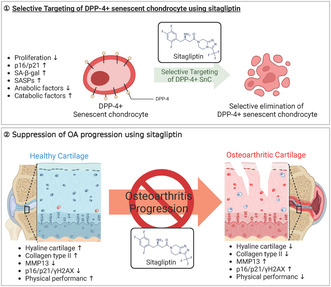

**Abbreviations**
ACLT: anterior cruciate ligament transection
CKIs: cyclin‐dependent kinase inhibitors
DMM: destabilization of the medial meniscus
DPP‐4: dipeptidyl peptidase‐4
ECM: extracellular matrix
FACS: fluorescence‐activated cell sorting
GEO: gene expression omnibus
NCBI: National Center for Biotechnology Information
NK: natural killer
OA: osteoarthritis
OARSI: osteoarthritis research society international
ROS: reactive oxygen species
RT‐qPCR: real‐time quantitative polymerase chain reaction
SASPs: senescence‐associated secretory phenotypes
SA‐β‐gal: senescence‐associated beta‐galactosidase
SBP: subchondral bone plate
SnCs: senescent chondrocytes

## 1 INTRODUCTION

Cellular senescence, triggered by stress signals, including telomere shortening, oxidative damage, oncogenic activation, mitochondrial dysfunction, and metabolic stress is a state of cell proliferation arrest (Di Micco et al., 2021; Watanabe et al., 2017). It is characterized by accumulation of senescence‐associated‐β galactosidase (SA‐β‐gal), enlarged morphology, emergence of senescence‐associated secretory phenotypes (SASPs), and expression of cyclin‐dependent kinase inhibitors (CKIs) (Di Micco et al., 2021; Watanabe et al., 2017). Senescent cells accumulate in many vertebrate tissues with age and contribute to age‐related diseases, such as cancer, cataracts, arthritis, and atherosclerosis (Childs et al., 2017; Watanabe et al., 2017). Selective elimination of p16^INK4a^‐positive senescent cells extended the lifespan of a transgenic mouse model of accelerated aging with restored vascular reactivity, stabilized atherosclerotic plaques, improved pulmonary function, alleviated osteoarthritis (OA), and ameliorated fatty liver disease (Baker et al., 2011). Thus, the increase in senescence that occurs with aging appears to play a major role in driving life‐limiting age‐related diseases (Baker et al., 2011; Di Micco et al., 2021).

Aging is a risk factor for OA, a chronic disease characterized by articular cartilage degradation, leading to pain, and physical disability (Han & Ro, 2021). Senescent chondrocytes (SnCs) were found in cartilage tissue isolated from patients undergoing joint replacement surgery (Jeon et al., 2017; Price et al., 2002). The clearance of senescent cells attenuates the development of post‐traumatic OA and creates a pro‐regenerative environment (Jeon et al., 2017). These reports indicate that SnCs play a causal role in cartilage degradation, and their subsequent increase induces loss of joint function in OA. However, no reported senolytics which specifically target SnCs have been developed, and the recent completed phase II trial of UBX0101 (NCT04129944) could not meet sufficient clinical efficacy. In addition, senescent cells may have cell type‐ and tissue‐specific regulations in their phenotypes and signaling mechanisms (Xu et al., 2022; Yousefzadeh et al., 2020). Hence, these reports indicate the importance of screening senescent chondrocyte‐specific markers and senolytic drugs that specifically target the senescence markers.

To develop or screen effective senolytics for the selective elimination of SnCs, it is necessary to understand senescent cell‐specific signaling mechanisms by separating them. However, most senescent cell‐specific markers are intracellular proteins and hence difficult to separate. Therefore, screening for cell‐surface factors that can serve as markers and therapeutic targets is important for selectively eliminating SnCs.

We aimed to identify a senescence marker that enables cell separation in a living state through Fluorescence‐activated cell sorting (FACS)‐based analysis, evaluate whether chondrocytes that express a selected marker show senescent and OA phenotypes, screen a novel senolytic candidate, and investigate the effect of targeting and removing SnCs expressing the selected cell surface marker in a rat OA model.

## 2 RESULTS

### 2.1 SnCs increase in OA cartilage

**FIGURE 1:**
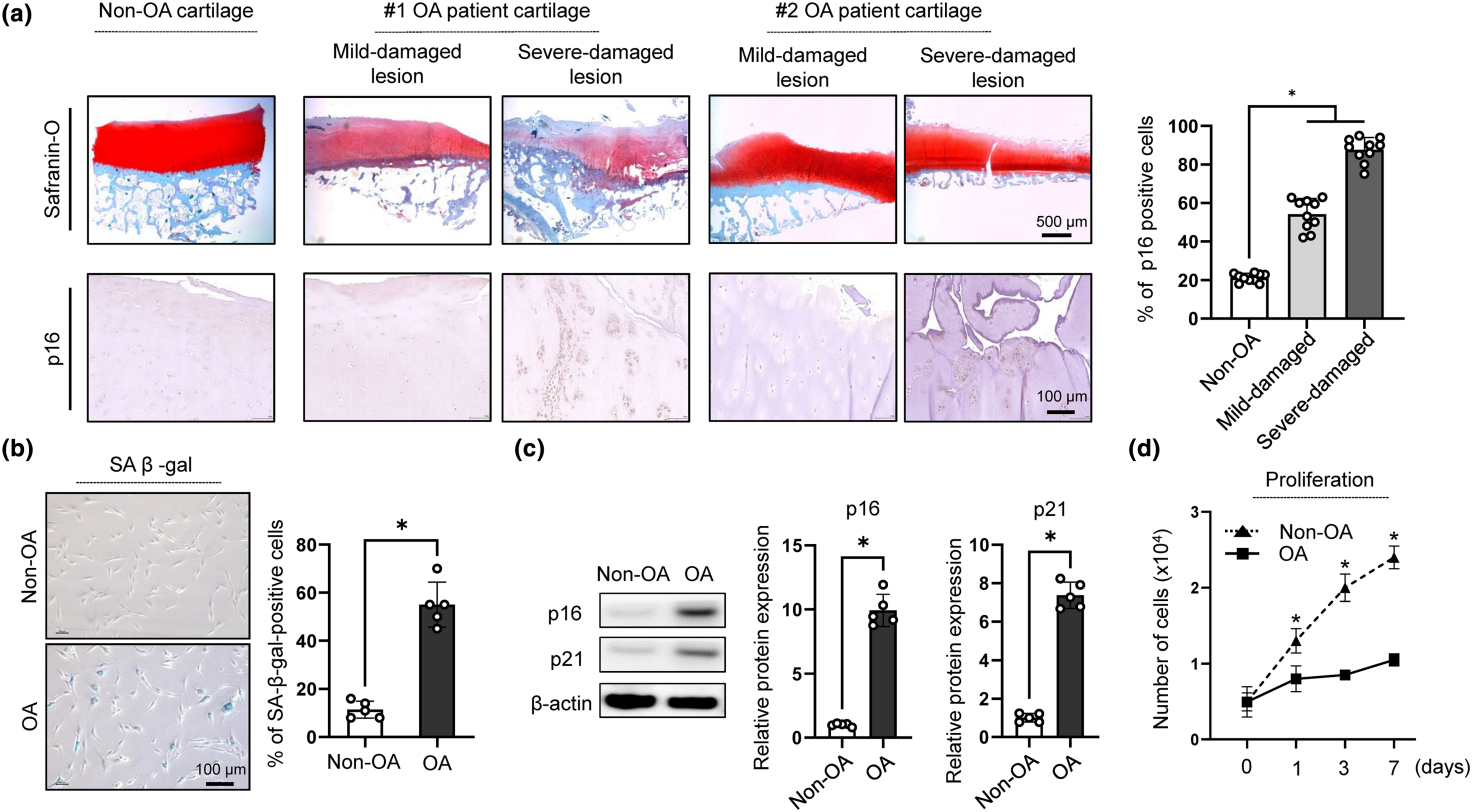
Increases in senescent chondrocytes in the cartilage of OA patients are closely related to cartilage damage. (a) (Left) Safranin‐O and IHC staining of p16 for Non‐OA and OA cartilage (severe‐ and mild‐damaged lesion), showing representative two donors. (Right) Quantification of p16 positive cells from Non‐OA and OA patients (*n* = 10). (b) (Left) SA β‐gal staining for Non‐OA and OA chondrocytes and (right) quantification of SA β‐gal positive cells (*n* = 5). (c) (Left) Representative image of western blot of p16 and p21 for chondrocytes from non‐OA and OA patients. (Right) Quantification of western blot analysis of p16 and p21 expression in non‐OA and OA chondrocytes (*n* = 5). (d) Cell counts of non‐OA and OA chondrocyte for 7 days to evaluate cell proliferation (*n* = 5). *p < 0.05.

To investigate the relationship between chondrocyte senescence and OA, we sought to detect SnCs in the human OA cartilage. The expression of p16, a prominent marker of cellular senescence, was significantly increased in chondrocytes from the OA cartilage (Figure 1a). The expression of p16 was positively associated with the severity of damaged cartilage, showing minimal expression in healthy cartilage and high intensity in severely damaged OA lesions. Subsequent senescence analysis revealed that approximately 57% of human OA chondrocytes stained positive for SA‐β‐gal (Figure 1b), and the expression of p16 and p21, senescence markers, was increased in cells from OA patients (Figure 1c). OA chondrocytes had decreased proliferative potential compared to non‐OA chondrocytes, indicating that OA chondrocytes underwent cell‐cycle arrest (Figure 1d). These results indicated that an increase in SnCs in the cartilage of patients was closely related to cartilage damage.

### 2.2 Screening for a surface factor selectively expressed on SnCs

**FIGURE 2:**
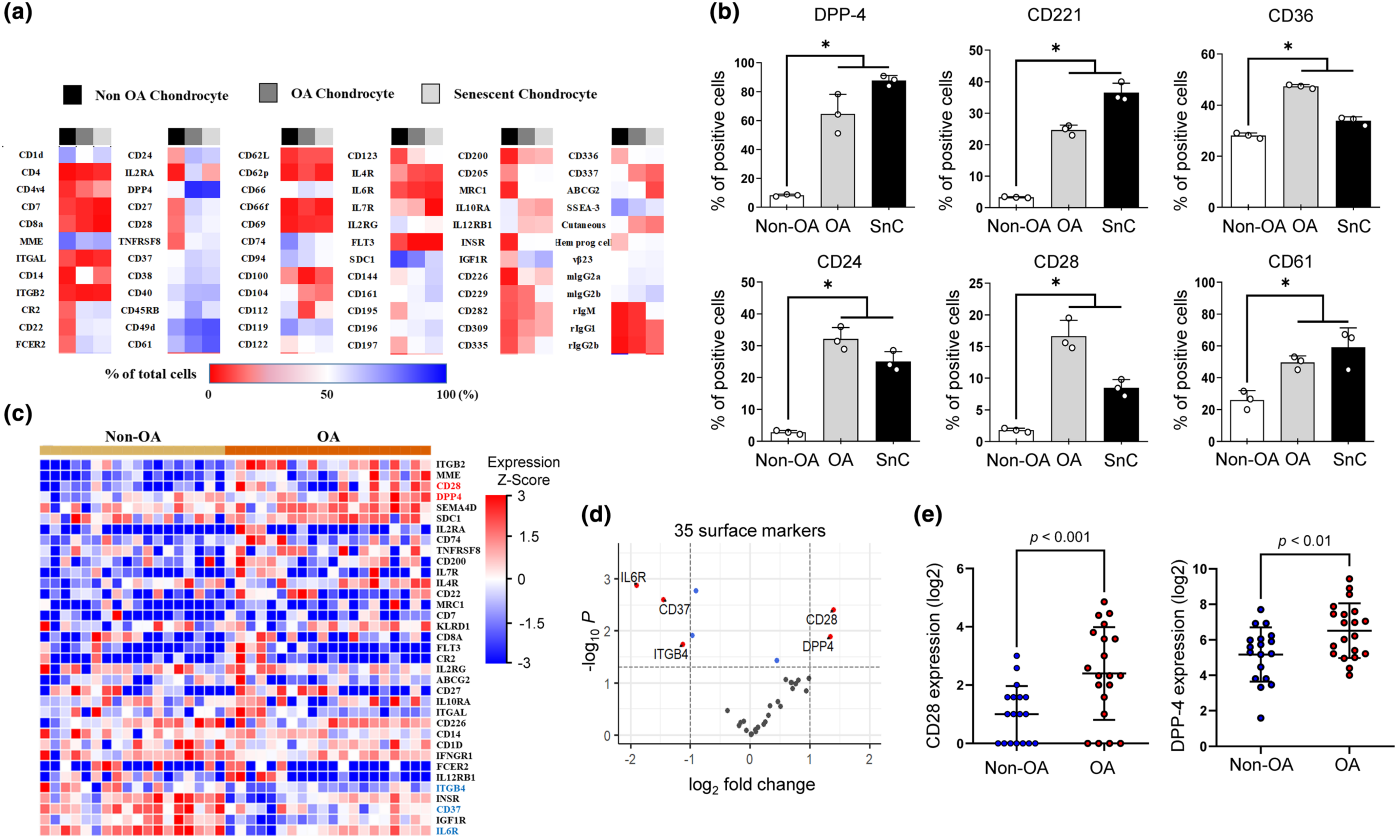
Screening for a surface factor selectively expressed on senescent and OA chondrocytes. (a) The expression heatmap of surface marker genes in non‐OA chondrocytes, OA chondrocytes and oxidative stress‐induced senescent chondrocytes (*n* = 3, data showing the average of the positive cell percentage from three donors). (b) Analysis of surface markers (DPP‐4, CD221, CD36, CD24, CD28 and CD61) for Non‐OA, OA and oxidative stress‐induced senescent chondrocytes (SnCs) (*n* = 3). (c) The expression heatmap of 35 surface marker genes in non‐OA (*n* = 18) and OA patients (*n* = 20) was obtained from the NCBI Gene Expression Omnibus (GEO) under the accession code GSE114007. (d) volcano plot of 35 surface markers in osteoarthritis patients, using fold‐differences and *p*‐values of the expression level, based on the student's t‐test between non‐OA patients and OA patients (the names of significantly regulated genes (log2 fold‐change >1 or < −1 and *p* < 0.05) are indicated) and (e) log2 expression of significantly upregulated genes (CD28 and DPP‐4) between patients with either non‐OA or OA. * *p* < 0.05.

To screen for surface markers that are selectively expressed in senescent OA chondrocytes, we set non‐senescent non‐OA chondrocytes (hereafter referred to non‐OA chondrocytes) as a control and performed flow cytometric screening of surface markers using Lyoplate to identify preferentially upregulated markers in human OA chondrocytes and H_2_O_2_‐induced SnCs. The results confirmed that 39 cell surface markers were increased in OA chondrocytes and oxidative stress‐induced SnCs in comparison with non‐OA chondrocytes (Figure 2a). Among the screened markers, six surface markers–dipeptidyl peptidase‐4 (DPP‐4); (CD26), CD221, CD36, CD24, CD28, and CD61 showed significant increases in both OA and SnCs, and DPP‐4 showed the highest increase in both OA and SnCs, with approximately 64.6% and 87.7%, respectively (Figure 2b). We cross‐validated the expression profiles of the surface markers listed in Figure 2a,b with non‐OA and OA patient transcriptome data from the NCBI GEO. Among the 39 surface markers shown in Figure 2a,b, 35 genes were matched to the transcriptome data, and CD28 and DPP‐4 were significantly upregulated in patients with OA (Figure 2c–e). In this regard, we selected DPP‐4 as the final senescent chondrocyte marker based on the results that DPP‐4 showed the greatest increase in both OA chondrocytes and SnCs (Figure 2b) and based on the cross‐validation analysis (Figure 2c–e).

**FIGURE 3:**
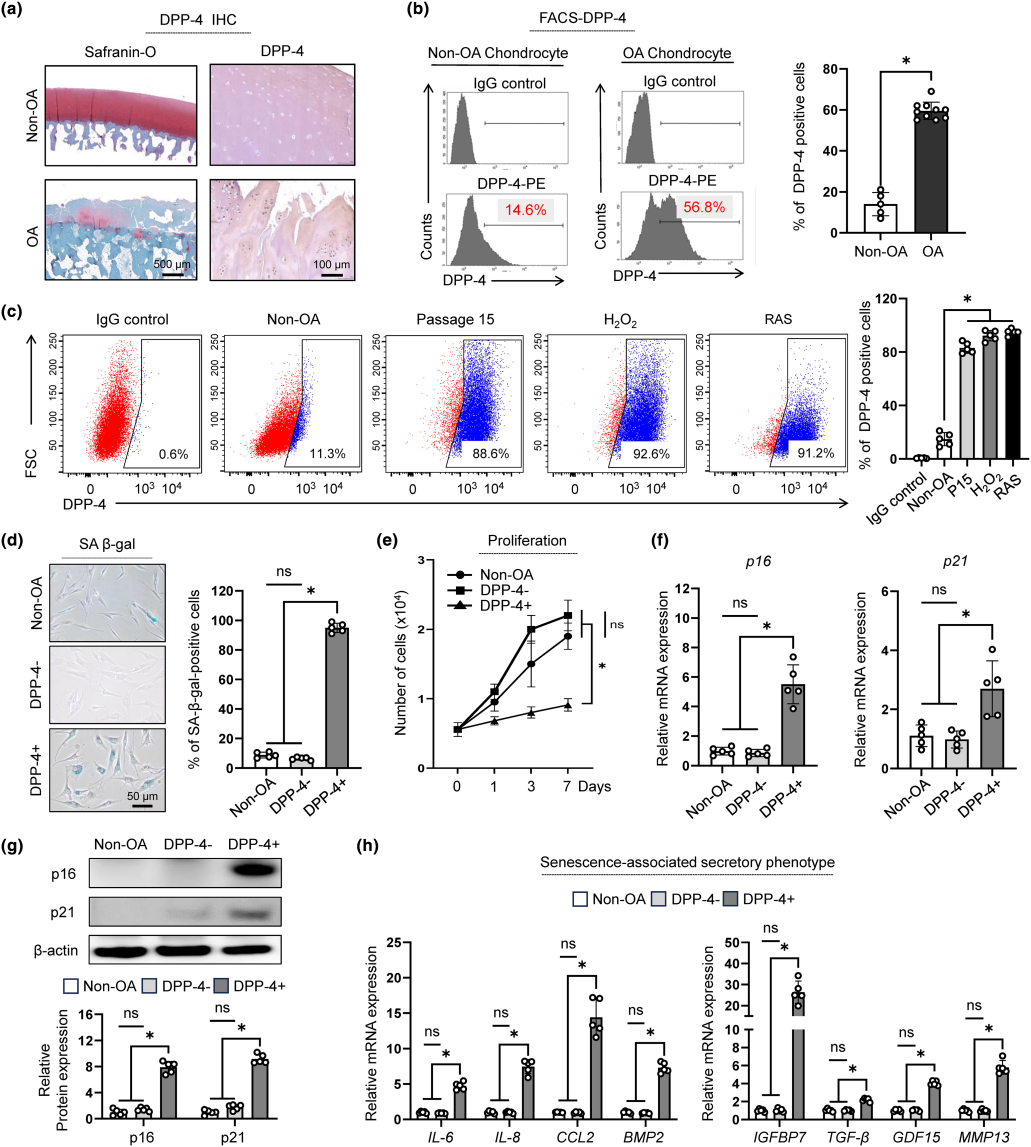
DPP‐4+ chondrocytes showed multiple senescent phenotypes. (a) Representative Safranin‐O and IHC staining of DPP‐4 for Non‐OA and OA cartilage. (b) (Left) Representative flow cytometry analysis of DPP‐4 expression from non‐OA and OA chondrocytes. (Right) Quantification of DPP‐4 positive cells determined by flow cytometry (*n* = 5 and *n* = 10, respectively). (c) (Left) Representative flow cytometry DPP‐4 (x‐axis) vs forward scatter (FSC: y‐axis) plot from IgG control, non‐OA, replicative (passage 15; P15), H_2_O_2_‐ and RAS‐induced senescent chondrocytes. (Right) Bar graph showing the percentage of DPP‐4 positive cells determined by flow cytometry (*n* = 5). (d) (Left) Representative SA β‐gal staining for non‐OA, DPP‐4‐ and DPP‐4+ chondrocytes. (Right) Quantification of SA β‐gal positive cells in non‐OA, DPP‐4‐ and DPP‐4+ chondrocytes (*n* = 5). (e) Cell counts of non‐OA, DPP‐4‐ and DPP‐4+ chondrocyte for 7 days to evaluate cell proliferation (*n* = 5). (f) The mRNA expression of p16 and p21 of non‐OA, DPP‐4‐ and DPP‐4+ chondrocytes using RT‐qPCR (*n* = 5). (g) (Top) Representative image of western blot analysis of p16 and p21 of DPP‐4‐ and DPP‐4+ chondrocytes. (Bottom) Quantification of western blot analysis of p16 and p21 expression in non‐OA, DPP‐4‐ and DPP‐4+ chondrocytes (*n* = 5). (h) The mRNA expression of SASPs (IL‐6, IL‐8, CCL2, BMP2, IGFBP7, TGF‐β, GDF15 and MMP13) in non‐OA, DPP‐4‐ and DPP‐4+ chondrocytes using RT‐qPCR. * *p* < 0.05.

To confirm these results, we evaluated the expression of DPP‐4 through tissue staining and FACS analysis. Immunohistochemical staining of DPP‐4 from patients exhibited increased DPP‐4 expression compared to non‐OA cartilage (Figure 3a). According to FACS analysis, approximately 57% of chondrocytes isolated from OA cartilage expressed DPP‐4, whereas only 14.6% of non‐OA chondrocytes expressed DPP‐4 (Figure 3b). We further investigated DPP‐4 expression in various conditions. Considering that chronological aging and high‐fat diet were reported to accumulate senescent cells (Schafer et al., 2016; Yousefzadeh et al., 2020), we asked whether DPP‐4 expression was upregulated along with p16 expression in aging‐ and high‐fat diet‐induced OA mice. Compared to young mice, old OA mice and high‐fat diet‐induced OA mice had significantly higher number of p16+ and DPP‐4+ SnCs (Data S1, Figure S1). To validate that DPP‐4 was expressed in various senescence‐induced in vitro models, we established three in vitro senescence induction model: replicative, oxidative stress (H_2_O_2_)‐ and oncogene (RAS)‐induced SnCs (Ashraf et al., 2016; Brandl et al., 2011; Serrano et al., 1997). All the senescence‐induced chondrocytes showed typical senescent cell phenotypes, including a significantly enlarged morphology, reduced cell growth, increased SA‐β‐gal staining, *p16*, *p21* and γH2AX expression (Data S1, Figure S2–S4). Consistent with the observed senescence phenotypes, we found that the proportion of DPP‐4+ chondrocytes significantly increased in replicative, H_2_O_2_‐ and RAS‐induced SnCs (Figure 3c). In addition, DPP‐4+ chondrocytes gradually increased at serial passages, and over 80% of chondrocytes were DPP‐4+ at passage 15 (Data S1, Figure S2F). These results prove that the observed trend of DPP‐4 expression aligned with the expression patterns of senescence phenotypes in replicative, H_2_O_2_‐ and RAS‐induced SnCs, indicating that DPP‐4 could be a senescence‐specific surface marker for SnCs.

### 2.3 DPP‐4‐positive chondrocytes show senescence phenotypes

We further asked whether senescence characteristics were specific to DPP‐4+ chondrocytes derived from human OA joints. Cell sorting was performed using anti‐DPP‐4 antibodies to separate DPP‐4+ and DPP‐4‐ chondrocyte populations from OA chondrocytes, which were then compared to non‐senescent non‐OA chondrocytes. The sorted DPP‐4+ chondrocytes showed cellular senescence phenotypes, with increased SA‐β‐gal positive cells, reduced growth rate, elevated mRNA and protein expression of the cell cycle arrest genes *p16* and *p21*, while the phenotypes of DPP‐4‐ chondrocytes were statistically nonsignificant to non‐OA chondrocytes (Figure 3d–g). Senescent cells also upregulate inflammatory cytokines, immune modulators, growth factors and proteases known as SASPs (Di Micco et al., 2021; Watanabe et al., 2017). Accordingly, we investigated whether DPP‐4+ chondrocytes expressed high levels of SASPs, compared to DPP‐4‐ and non‐OA chondrocytes. As a result, IL‐6, IL‐8, CCL2, BMP2, IGFBP7, TGF‐β, GDF15 and MMP13, which were well studied for inducing paracrine senescence (Acosta et al., 2013; Guo et al., 2019; Lopes‐Paciencia et al., 2019; Zhang et al., 2022), were significantly upregulated in DPP‐4+ chondrocytes (Figure 3h). Taken together, DPP‐4+ chondrocytes were SnCs which exhibited multiple senescent phenotypes.

### 2.4 DPP‐4‐positive chondrocytes show increases in OA‐associated phenotypes

**FIGURE 4:**
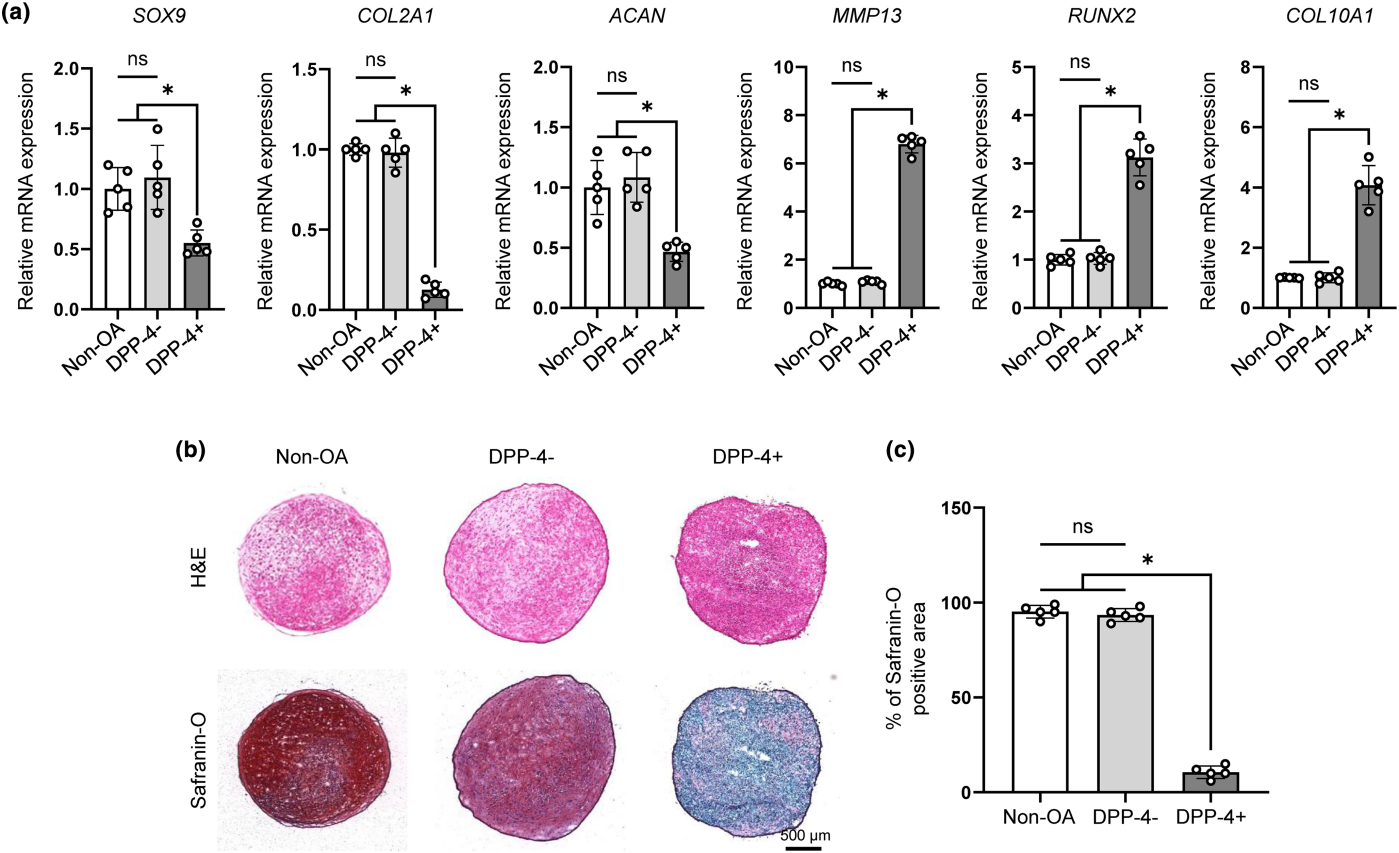
DPP‐4+ chondrocytes had OA phenotypes. (a) The mRNA expression of chondrocyte markers (SOX9, COL2A1, ACAN), hypertrophic chondrocyte markers (RUNX2, COL10A1) and catabolic markers (MMP13) in non‐OA, DPP‐4‐ and DPP‐4+ chondrocytes (*n* = 5). (b) Representative H&E and safranin‐o staining for pellets generated from non‐OA, DPP‐4‐ and DPP‐4+ chondrocytes and (c) Quantification of safranin‐O positive area of non‐OA, DPP‐4‐ and DPP‐4+ pellets (*n* = 5). * *p* < 0.05.

To investigate whether DPP‐4+ chondrocytes could contribute to the loss of cartilage in OA, we examined OA‐associated catabolic signaling in DPP‐4+ chondrocytes. In comparison with DPP‐4‐ and non‐OA chondrocytes, the mRNA levels of chondrogenesis‐related genes (*SOX9, COL2A1* and *ACAN*) were significantly decreased in DPP‐4+ chondrocytes, in contrast to the expression levels of hypertrophic chondrocyte‐related genes (*RUNX2* and *COL10A1*) and catabolic markers (*MMP13*) which were strongly increased in DPP‐4+ chondrocytes (Figure 4a **)**. To evaluate the synthesis of proteoglycan in DPP‐4+ chondrocytes, we generated 3D pellets using non‐OA, DPP‐4‐, or DPP‐4+ chondrocytes. The pellet culture of DPP‐4+ chondrocytes showed reduced safranin‐O staining of proteoglycans, suggesting that the expression of DPP‐4 correlated with the loss of hyaline cartilage extracellular matrix (ECM) (Figure 4b,c). These findings suggest that DPP‐4+ SnCs can induce cartilage destruction by upregulating catabolic and hypertrophic factors and decreasing hyaline ECM synthesis.

### 2.5 Sitagliptin could specifically and effectively target DPP‐4 positive SnCs both in vitro and in vivo

**FIGURE 5:**
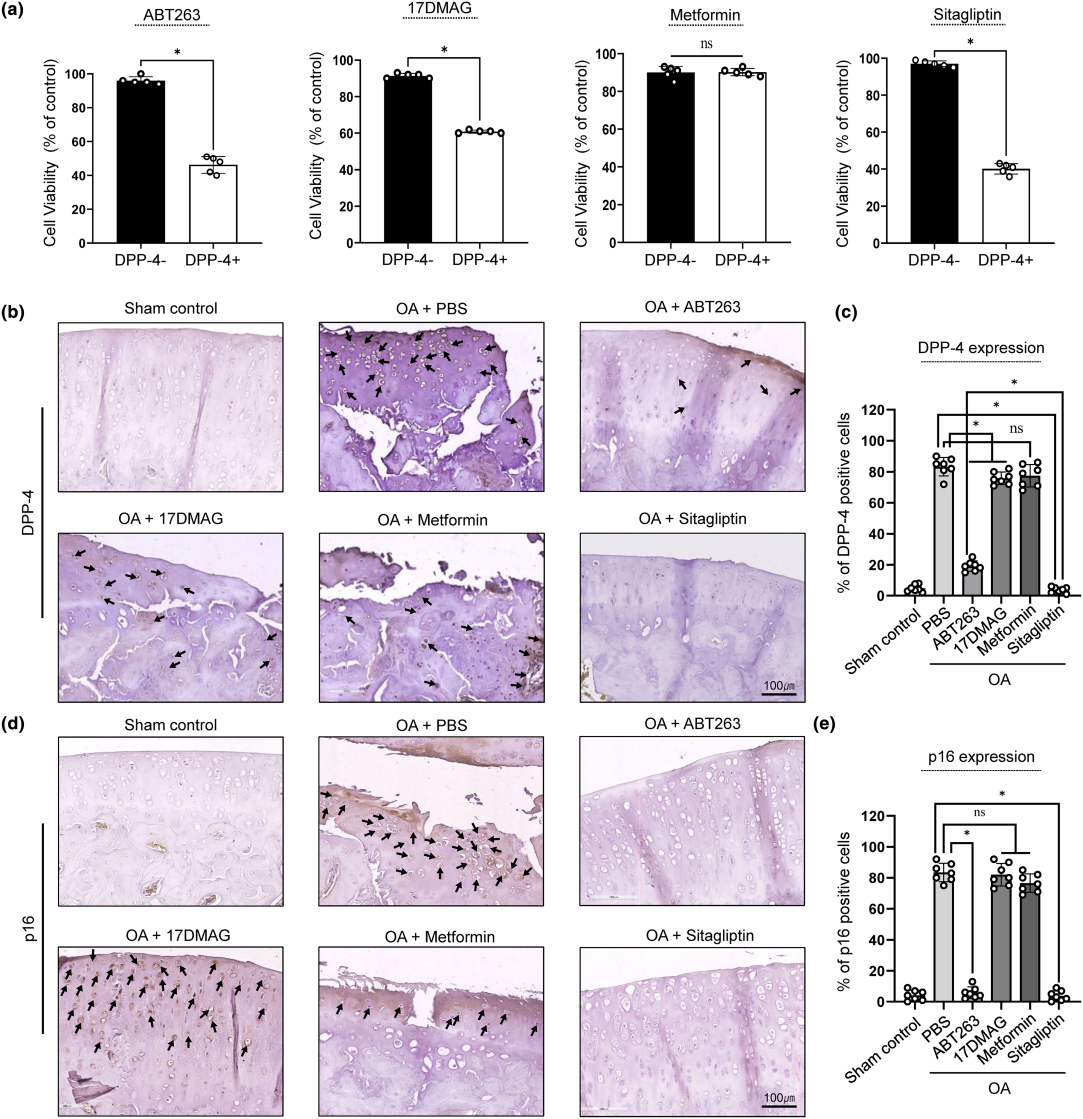
Sitagliptin could specifically and effectively target DPP‐4 positive chondrocytes. (a) Evaluation of cell viability of DPP‐4‐ and DPP‐4+ chondrocytes upon treatment of 20 μM ABT263, 100 nM 17DMAG, 20 μM metformin or 5 μM sitagliptin (*n* = 5). (b, d) Representative histological analysis using IHC for DPP‐4 and p16 staining to evaluate in vivo senolytic effects of ABT263, 17DMAG, metformin and sitagliptin and (c, e) Quantification of DPP‐4 and p16 positive cells (*n* = 7 per group). * *p* < 0.05.

Previous studies have shown that ABT263 (a pan‐Bcl inhibitor), 17DMAG (a heat shock protein 90 [HSP90] inhibitor), and metformin (an AMPK regulator) exert senolytic and senomorphic effects on senescent cells (Gasek et al., 2021). To determine whether DPP‐4+ chondrocytes are susceptible to these senolytics, DPP‐4‐ and DPP‐4+ chondrocytes were treated in vitro. There was no significant difference in cell viability between DPP‐4‐ and DPP‐4+ chondrocytes treated with metformin (Figure 5a). Treatment with ABT263 or 17DMAG decreased the viability of DPP‐4+ chondrocytes without significantly altering the viability of DPP‐4‐ chondrocytes, suggesting that DPP‐4+ chondrocytes are susceptible to senolytics (Figure 5a). To further evaluate whether DPP‐4+ chondrocytes could be eliminated by treating with DPP‐4 inhibitors and to select effective DPP‐4 inhibitor, we treated chondrocytes with sitagliptin, saxagliptin, and gemigliptin to DPP‐4‐ and DPP‐4+ chondrocytes. The viability of DPP‐4+ chondrocytes significantly decreased without altering the viability of DPP‐4‐ chondrocytes (Figure 5a, Data S1, Figure S5A). Previous reports revealed that sitagliptin and gemigliptin have higher selectivity toward DPP‐4 against other DPP families (DPP‐8 and DPP‐9) than saxagliptin (Chen et al., 2015; Kim et al., 2016). Although gemigliptin had the highest selectivity and inhibitory potency, according to the previous report (Kim et al., 2016), our in vitro analysis showed that sitagliptin was the most effective at targeting DPP‐4+ SnCs (Data S1, Figure S5B). Therefore, we selected sitagliptin for validation of its in vivo efficacy. A DMM‐induced rat OA model was established and injected with PBS, ABT263, 17DMAG, metformin, or sitagliptin into the rat knee joints. Surgical OA induction resulted in the accumulation of DPP‐4+ SnCs in the articular cartilage, with apparent increase in p16, p21 and γH2AX expression (Figures 5b–e and Data S1, Figure S6). Intra‐articular injection of ABT263 or 17DMAG significantly decreased the number of DPP‐4+ SnCs compared to PBS, whereas the metformin injection group had no effect in vivo (Figures 5b–e and Data S1, Figure S6). Remarkably, sitagliptin eliminated DPP‐4+ SnCs most effectively, compared to the PBS and ABT263 groups. The clearance of DPP‐4+ SnCs by sitagliptin was confirmed by significantly decreased DPP‐4‐, p16‐, p21‐ and γH2AX‐positive chondrocytes (Figure 5b–e and Data S1, Figure S6). These results suggest that sitagliptin could serve as a senolytic drug that selectively and effectively targets DPP‐4+ senescence chondrocytes.

### 2.6 Selective targeting of DPP‐4‐positive chondrocytes via intra‐articular injection of sitagliptin prevents OA development in a rat DMM model

**FIGURE 6:**
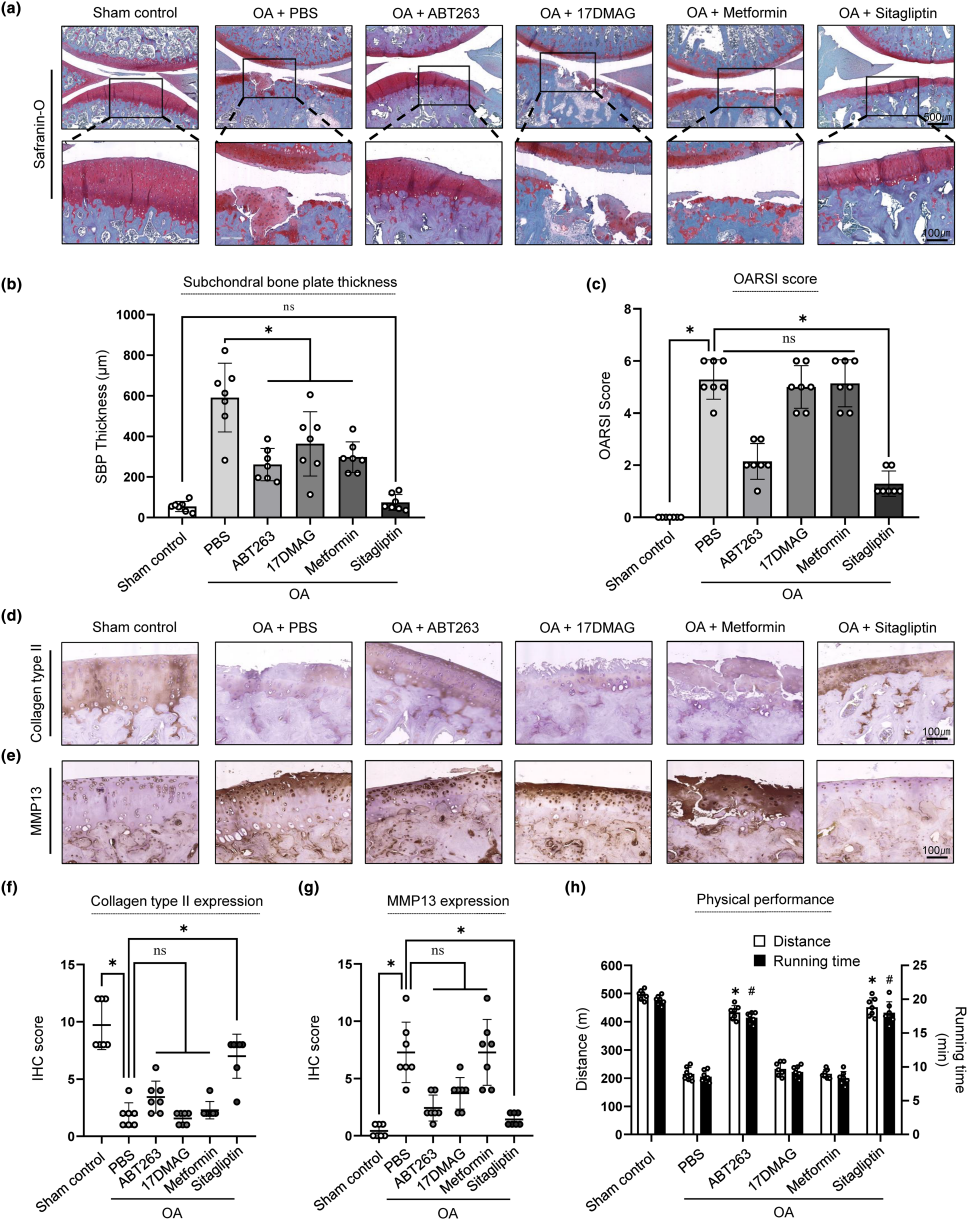
Intra‐articular injection of the DPP‐4‐inhibitor sitagliptin prevented OA development in the rat DMM model. (a) Representative histological analysis using safranin‐O to evaluate OA progression in sham control and DMM rats after PBS, ABT263, 17DMAG, metformin or sitagliptin injection, (b) quantification of subchondral bone plate thickness of sham control and DMM rats after PBS, ABT263, 17DMAG, metformin or sitagliptin injection and (c) scoring of OA using OARSI grading system in sham control and DMM‐induced OA rat that had undergone intra‐articular injection of PBS, ABT263, 17DMAG, metformin or sitagliptin (*n* = 7). Representative collagen type II (d) and MMP13 (e) IHC staining images of sham control and DMM rats injected with PBS, ABT263, 17DMAG, metformin or sitagliptin and (f, g) quantification collagen type II and MMP13, respectively, using scoring system (*n* = 7). (h) Treadmill running distance and time (*n* = 7), and # and * indicate *p* < 0.05 when compared to PBS groups, respectively. *p < 0.05.

Based on a previous report that the elimination of SnCs suppressed OA progression (Jeon et al., 2017), we investigated whether selective targeting of DPP‐4+ chondrocytes, as shown in Figure 5b had protective effects on OA progression. Eight weeks after surgery, severe cartilage destruction with thickening of the subchondral bone plate (SBP) and significantly increased OARSI score were observed in the PBS‐injected group (Figure 6a–c). Intra‐articular injection of ABT263 led to intact articular cartilage and a significant reduction in SBP thickness eight weeks after injection, indicating that ABT263 prevented OA progression, as previously reported (Yang et al., 2020). Although treatment with 17DMAG or metformin improved SBP thickening, neither 17DMAG nor metformin prevented OA development, as confirmed by safranin‐O staining, which showed that the cartilage remained severely destroyed with a loss of proteoglycans and a high OARSI score (Figure 6a–c). In contrast, sitagliptin injection resulted in intact articular cartilage with increased proteoglycan synthesis, ameliorated cartilage damage and improved SBP thickness, with no significant difference compared to the sham control. Notably, only the group injected with sitagliptin showed significant decrease in OARSI score whereas other senolytic treatment groups were not statistically significant, compared to the PBS injected group (Figure 6c). Collectively, these results suggest that selective inhibition of DPP‐4+ chondrocytes using sitagliptin could effectively ameliorate OA progression in a DMM‐induced rat OA model.

It has been well studied that OA chondrocytes show downregulated hyaline ECM molecules and upregulated matrix‐degrading enzymes (Troeberg & Nagase, 2012), we further assessed whether these senolytic treatments could regulate these phenotypes. We found that the PBS‐injected DMM‐induced OA model had significantly decreased collagen type II expression and upregulated MMP13 expression compared to the sham control. (Figure 6d–g). Neither collagen type II nor MMP13 expression was significantly affected by the intra‐articular injection of senolytics, including ABT263, 17DMAG, or metformin. However, collagen type II and MMP13 expression improved only in the sitagliptin‐treated group. Finally, we evaluated whether histologically confirmed prevention of OA could lead to improvements in physical performance. The groups treated with ABT263 or sitagliptin injection had significantly improved physical functions, as confirmed by longer treadmill running distance and time (Figure 6h). These results indicate that selective targeting of DPP‐4+ chondrocytes by inhibiting DPP‐4 prevents cartilage degradation by reducing matrix catabolic factors, upregulating hyaline ECM components, and recovering physical abilities.

## 3 DISCUSSION

Aging is a risk factor for the development of OA, a chronic disease characterized by articular cartilage degeneration, leading to pain and physical disability (Han & Ro, 2021). Previous reports have revealed that SnCs are present in cartilage tissues isolated from patients with OA (Liu et al., 2022; Yang et al., 2020). SnCs accumulate in the articular cartilage and synovium after anterior cruciate ligament transection (ACLT), and the selective elimination of these cells attenuates the development of post‐traumatic OA, reduces pain, and increases cartilage development (Jeon et al., 2017). Our results also revealed that the cartilage of patients with OA had a significant proportion of SnCs, as confirmed by increased SA‐β‐gal, p16, and p21 expression. It has been reported that the selective removal of SnCs from in vitro cultures of chondrocytes isolated from patients with OA results in a reduction in the expression of senescent and inflammatory markers and an increased expression of hyaline cartilage ECM proteins (Yang et al., 2020). Despite the significant role of SnCs in OA development, there are currently no senolytics which specifically target SnCs, resulting in insufficient clinical efficacy of senolytics, such as UBX0101 (NCT04129944). This have highlighted the importance of developing chondrocyte‐specific senolytics, but specific elimination of these cells is challenging because of the lack of senescent chondrocyte‐specific marker, the intracellular location of well‐known senescence markers and intracellular delivery of senolytics.

DPP‐4 is expressed ubiquitously in many endothelial and epithelial tissues, including, but not limited to, the kidneys, liver, lungs, intestine, and immune cells (e.g., T cells, activated B cells, activated natural killer [NK] cells, and myeloid cells). Kim et al. reported that DPP‐4 is selectively expressed on the surface of senescent but not proliferating human diploid fibroblasts (Kim et al., 2017). Here, we found that SnCs in OA cartilage and replicative, H_2_O_2_‐ and RAS‐induced SnCs strongly upregulated DPP‐4. We confirmed that DPP‐4 could be a senescence marker for senescent OA chondrocytes by analyzing SA‐β‐gal expression, proliferation, senescence marker expression and SASP expression in DPP‐4+ chondrocytes. These SnCs had altered phenotypes with decreased anabolic factors (*COL2A1, ACAN* and *SOX9*) and safranin‐O staining, and increased catabolic (*MMP13*) and hypertrophic markers (*RUNX2 and COL10A1*). There was a small proportion of DPP‐4+ chondrocytes existed in non‐OA cartilage (approximately 15% in Figure 3b). A recent study revealed that p16‐positive fibroblasts with a certain senescent phenotype could have beneficial effects with respect to the reparative niche in the lungs by increasing the sensitivity to inflammation and enhancing epithelial regeneration (Reyes et al., 2022). Further study is required to investigate whether the small proportion of DPP‐4+ chondrocytes in non‐OA cartilage observed in our study may have contributed to cartilage homeostasis.

Previous study has found a significant increase of DPP‐4 expression in OA cartilage, and DPP‐4 inhibition protected cartilage from IL‐1 + oncostatin M‐mediated collagen breakdown (Patel, 2011). Recent study also showed that chondrocytes expressing DPP‐4 had multiple senescence features and were associated with radiographic progression in OA (Chen et al., 2023). Furthermore, cytokine, such as IL‐1β and TNF‐α, −induced chondrocytes also exhibited senescence phenotypes with upregulation of DPP‐4 expression, and inhibition or knockdown of DPP‐4 using DPP‐4 inhibitor or siRNA in these chondrocytes were reported to alleviate senescent chondrocyte phenotypes (Bi et al., 2019; Mohetaer et al., 2018; Wang et al., 2020). Although cytokines have been reported to be involved in the pathological development of OA, there are limitations, considering that the onset and progression of OA and phenotypical change of non‐OA to OA chondrocytes are not induced by a single stimulation by cytokines but progresses in a complex manner due to the combination of several factors (Yao et al., 2023). In addition, these studies concentrated on in vitro analysis using cytokine‐stimulated chondrocytes or evaluated the effect of DPP‐4 inhibitor on OA protection through oral administration, which is unclear whether the effect of DPP‐4 inhibitor is indirect due to inhibition of soluble DPP‐4 in plasma or direct effect on DPP‐4+ chondrocytes. Supporting these previous papers, the present study proved an increase of DPP‐4 expression in cartilage and chondrocytes from OA patients without any additional induction process, and the upregulation of DPP‐4 is specific to SnCs, confirmed in various senescent chondrocyte models, including replicative, oxidative stress‐ and oncogene‐induced senescence model and evaluation of senescence phenotypes in DPP‐4‐sorted OA chondrocytes. The effect of DPP‐4 inhibition was shown to be DPP‐4+ chondrocyte specific as a sitagliptin treatment affected DPP‐4+ chondrocytes, but not DPP‐4‐ chondrocytes. Furthermore, we also showed that DPP‐4 expression was significantly upregulated in a DMM‐induced OA rat cartilage and that intra‐articular injection of sitagliptin could selectively and effectively eliminate DPP‐4+ SnCs, resulting in protection from OA progression in a DMM‐induced OA rat. Considering that p16‐positive SnCs were reported to be detected 3 days after surgical induction of OA and peak at 2 weeks (Jeon et al., 2017), the senolytic effect of sitagliptin was shown to eliminate DPP‐4+ chondrocytes, rather than by suppressing the conversion of healthy chondrocytes into SnCs. Furthermore, sitagliptin treatment in the posttraumatic OA model had the greatest therapeutic effect compared to other senolytics. It is interesting to note that a significant increase in DPP‐4+ SnCs was also found in animals with OA induced by aging and high‐fat dietary conditions, similar to that in surgically induced young OA animals. This result indicates that DPP‐4+ SnCs accumulate due to various factors and that sitagliptin may be effective in the treatment of OA induced by various status of an individual.

Senolytics, such as 17DMAG and metformin, which target key proteins involved in apoptosis (Gasek et al., 2021) have clear limitations. For example, HSP90, a target for 17DMAG, is known to contribute to stabilization of the client protein implicated in apoptosis and regulate the SASP and SA‐β‐gal activity in senescent retinal pigment epithelial cells (Chen et al., 2021). However, a decrease in *HSP90* mRNA expression was observed in OA compared to normal cartilage (Boehm et al., 2007), and basal HSP90 protein levels in cartilage declined during aging (Smith et al., 2006), which may lead to insufficient efficacy in our OA model. Metformin directly or indirectly activates AMPK, thereby lowering glucose production (Rena et al., 2017) and induces apoptosis through AMPK dependent (Leclerc et al., 2013) and independent (Samuel et al., 2017) pathways. Furthermore, metformin has a senostatic function that does not eliminate senescent cells but regulates SASP regulatory network via modulating NF‐κB (Kim et al., 2023; Schoetz et al., 2021). Critical problem with both 17DMAG and metformin arises that their target molecules also have non‐senescence‐related functions; HSP90 plays essential role in controlling cell cycle and survival (Jackson, 2012); AMPK signaling pathway coordinates cell growth and metabolism reprogramming (Mihaylova & Shaw, 2011); NF‐κB plays a major role in regulating acute inflammatory response and immune response (Chien et al., 2011). Collectively, these could result in broad effects rather than specific senolytic effects and lead to a lower effectiveness in our OA model. Our results indicate that the efficacy of sitagliptin in DMM‐induced OA joint was probably due to its specificity to selectively target DPP‐4+ SnCs, providing the first evidence that sitagliptin could be a potential OA medication that may overcome the current limitations of senolytic drugs.

Based on the results of DPP‐4 inhibitors selectively depleting DPP‐4+ chondrocytes, we have revealed that DPP‐4 could serve as a functional molecule in OA chondrocytes, but the exact mechanisms by which DPP‐4 expression leads to chondrocyte senescence or DPP‐4 upregulation and its downstream pathways are not fully elucidated. The regulatory mechanism of DPP‐4 in senescent chondrocyte could be inferred from previous studies. Overexpression of DPP‐4 elicited senescence phenotypes in fibroblasts (Kim et al., 2017), and knockdown of DPP‐4 ameliorated senescence phenotypes, confirmed by decreased p53, p21 and p16 expression and increased proliferation in fibroblasts and chondrocytes (Kim et al., 2017; Wang et al., 2020). Furthermore, in hepatocytes, recombinant DPP‐4 treatment increased cellular reactive oxygen species (ROS) production and NF‐kB activation while sitagliptin treatment alleviated these effects (Wang et al., 2021). DPP‐4 promoter region contains consensus sites for various transcription factors, such as NF‐kB, which could thereby regulate DPP‐4 expression (Böhm et al., 1995). Form these studies, it is conceivable that accumulation of stress in cartilage during aging would activate NF‐kB, which then increases expression of DPP‐4 in chondrocytes. Increased DPP‐4 expression might produce ROS and further activate NF‐kB. This may create a vicious cycle where accumulated stress in cartilage during aging activates NF‐kB/DPP‐4/oxidative stress axis, consequently leading to chondrocyte senescence and OA exacerbation. Whether these findings from other models could be apply to the mechanisms of senescent chondrocyte induction and accumulation during OA progression would require further studies.

In summary, using cell surface screening and transcriptome analysis, we verified that DPP‐4 is a cell surface marker for SnCs in OA cartilage. DPP‐4+ chondrocytes showed typical senescent and OA phenotypes. We selected previously reported senolytics (ABT263, 17DMAG, and metformin) and sitagliptin to assess their effects on the removal of DPP‐4+ chondrocytes. Among the three senolytics and sitagliptin tested in vivo, intra‐articular sitagliptin injection was most effective in targeting DPP‐4+ chondrocytes and preventing OA progression. Thus, selective targeting of DPP‐4+ chondrocytes using sitagliptin could be a promising strategy to prevent OA progression.

## 4 MATERIALS AND METHODS

### 4.1 Cell isolation and culture from human osteoarthritic joints

Human osteoarthritic cartilage from 10 patients undergoing total knee arthroplasty and non‐osteoarthritic cartilage from 10 patients who underwent tumor removal were obtained. This study was approved by our Institutional Review Board (No. H‐1907‐028‐1045, No. 0902–021‐271) and the patients provided informed consent for the donation of tissues for research purposes. Cartilage was minced and digested to collect chondrocytes. Isolated chondrocytes were seeded at a density of 1 × 10^6^ in 150 mm dishes and were cultured in high‐glucose DMEM containing 10% fetal bovine serum (FBS, Gibco, 16,000,044) and 1% antibiotic‐antimycotic solution at 37°C with 5% CO_2_. Chondrocytes underwent passaging upon reaching approximately 80% confluence. Non‐OA chondrocytes used in all the in vitro experiments were passage 2 (Data S1).

### 4.2 Chondrogenesis using pellet culture

Unsorted, DPP‐4 negative (DPP‐4‐) and DPP‐4+ chondrocytes were centrifuged at 1500 rpm for 5 min to obtain cell pellets, which were then cultured in a chondrogenic medium. The chondrocyte pellets underwent chondrogenesis for up to 21 days. The medium was refreshed every 3–4 days (Data S1).

### 4.3 Establishment of replicative, oxidative stress‐ and RAS‐induced SnCs

Non‐OA chondrocytes (passage 2) were seeded in 150 mm culture plate at a density of 1 × 10^6^ in high‐glucose DMEM containing 10% FBS and 1% antibiotic‐antimycotic solution at 37°C with 5% CO_2_. When chondrocytes have proliferated to confluences approximately 80%, chondrocytes were trypsinized, counted and seeded again at 1 × 10^6^ cells/150 mm dish. This process was repeated until reaching passage 15.

Non‐OA Chondrocytes were pretreated with serum‐free medium for 1 h, followed by treatment with 200 μM hydrogen peroxide (H_2_O_2_) for 16 h. The H_2_O_2_‐containing medium was removed after 16 h and incubated with fresh cell culture medium for 24 h. This process was repeated four times every 3 days.

Non‐OA chondrocytes were seeded in a 6‐well culture plate at a density of 5 × 10^4^ cells/well. The medium was changed with 1.0 mL of fresh complete medium and 0.5 mL of the respective viral preparation was added. The cationic polymer hexadimethrine bromide (polybrene) was added to each flask to reach a final concentration of 10 μg/mL. For selection of stable chondrocytes expressing RAS, the culture medium was changed with fresh medium containing 150 μg/mL G418 (Gibco, 10,131,035). After establishment of these senescence induction model, the SnCs were subjected to RT‐qPCR and SA‐β‐gal analysis (Data S1).

### 4.4 Cell surface marker screening by flow cytometry using BD lyoplate

BD Lyoplate human cell surface marker screening panel (BD Biosciences, 560,747) was used to screen senescent chondrocyte‐specific markers. The kit contained 242 purified monoclonal antibodies against cell surface markers. The assay was performed on three donors per group according to the manufacturer's instructions. Fluorescence was measured with a BD FACSCanto II cytometer on 10,000 cells using the FACSDiva software (See Data S1).

### 4.5 Transcriptomic analysis of surface markers in OA patients

Non‐OA and OA patient transcriptome data were downloaded from the National Center for Biotechnology Information (NCBI) Gene Expression Omnibus (GEO) under the accession code GSE114007 (Fisch et al., 2018) [10]. Probe IDs were mapped to gene symbols using the mapIds function in the R AnnotationDBI package (v1.52.0). Probes with the maximum mean expression levels across samples were collapsed into genes for subsequent analyses. Morpheus (https://software.broadinstitute.org/morpheus/) was used to generate the expression heat maps of the surface factors. A volcano plot was generated using the Enhanced Volcano package (v1.14.0) in R Studio.

### 4.6 Cell sorting and flow cytometric analysis

Cartilage tissue from OA patients was finely minced, digested with 0.025% collagenase in high‐glucose DMEM containing 1% antibiotic‐antimycotic solution and incubated overnight at 37°C with 5% CO_2_. The digested OA chondrocytes was filtered through a 70 μm strainer and labeled with DPP‐4‐PE (BD Pharmingen, 555,437) or mIgG‐PE (BioLegend, 405,307) for 30 min at 4°C in the dark. OA chondrocytes were sorted according to side scattering and PE intensity (Data S1).

### 4.7 Cell viability assay

Senolytics (20 μM ABT263, 100 nM 17DMAG, and 20 μM metformin) and 5 μM gliptins (sitagliptin, saxagliptin, and gemigliptin) were treated for 3 days. The cell viability assay was performed by counting the number of cells after trypan blue staining (See Data S1).

### 4.8 SA‐β‐gal staining

A SA‐β‐gal staining kit was used to evaluate SA‐β‐gal activity. After fixation, the cells were incubated overnight with the staining mixture at 37°C and were then observed under an inverted microscope. (Data S1).

### 4.9 Real time quantitative polymerase chain reaction (RT‐qPCR) analysis

RNA extraction, reverse transcription, and RT‐qPCR were conducted using the RNeasy mini kit (Qiagen, 74,104), cDNA kit (TaKaRa, 639,543), and TaqMan gene expression assay kits (Applied Biosystems, 4,331,182), respectively. TaqMan probes were purchased from Thermo Fisher (listed in Table S1). The 2^−ΔΔCT^ method was used to evaluate the relative expression of each target gene. (See Data S1).

### 4.10 Western blot analysis

Proteins were collected by lysing chondrocytes using RIPA buffer (Thermo Fisher, 89,900), separated using SDS‐PAGE and transferred to PVDF membranes. After blocking procedure, the membranes were probed with primary antibodies against p16 (Cell Signaling Technology, 80772S), p21 (Cell Signaling Technology, #2947), and actin (Sigma‐Aldrich, A5441) overnight. Proteins were visualized using secondary antibodies conjugated to horseradish peroxidase (Invitrogen, 31,460 [rabbit], 31,437 [mouse]) (See Data S1).

### 4.11 Immunofluorescence staining

2 × 104 chondrocytes were seeded in Lab‐Tek II 4‐well chamber slide (Thermo Fisher Scientific, 154,526). Chondrocytes were fixed using BD Cytofix/Cytoperm (BD bioscience, 554,722) for 30 min at 4°C, blocked using 3% BSA in PBS for 30 min and incubated with primary antibody γH2AX (Cell signaling Technology, #9718) diluted (1:200) in PBS overnight at 4°C. Chondrocytes were visualized using confocal microscopy (Leica STELLARIS 8) after DAPI staining and mounting using Antifade Mounting Medium with DAPI (Vector Laboratories, H‐1200). (See Data S1).

### 4.12 Old mice and high‐fat diet mice

For old mice, 68‐week‐old mice were purchased from Laboratory Animal Resource and Research Center of Korea Research Institute of Bioscience and Biotechnology (LARRC‐KRIBB, South Korea). For high‐fat diet mice, 24‐week‐old diet‐induced‐obesity mice were purchased from Central Lab. Animal Inc. (South Korea). Mice were sacrificed after finishing acclimatization. Harvested knee were subjected to immunohistochemistry staining of DPP‐4 and p16. (Data S1).

### 4.13 Surgical OA induction and motility test using treadmill

All experimental procedures related to the animal models were approved by the Institutional Animal Care and Use Committee (IACUC, No. 22–0038). Destabilization of the medial meniscus (DMM) was performed in male Wistar rats (4 months; *n* = 7 per group). A sham surgery (control group) was conducted. All the surgeries were performed on the same day. Seven days after DMM surgery, treatment groups including PBS (80 μL vehicle), ABT263, 17DMAG, metformin, and sitagliptin (5 mM/80 μL, each) were randomly allocated. For the injection groups, intra‐articular injections were administered once every 3 days for 7 weeks. Eight weeks after surgery, the rats were tested for motility on a treadmill, and the knee joints were collected for histological analysis. (Data S1).

### 4.14 Histology and immunohistochemistry

Human OA and non‐OA joint tissues, and rat knee joints were fixed in paraformaldehyde, dehydrated with ethanol, and embedded in paraffin. These sections were stained with safranin O/fast green. For immunostaining, paraffin‐embedded sections were deparaffinized with xylene and dehydrated. The sections were then treated with 3% H_2_O_2_, processed with hyaluronidase, and incubated with 10% FBS to block nonspecific binding. The sections were incubated with primary antibody p16 (Abcam, ab54210), p21 (ABClonal, A19094), γH2AX (Cell signaling Technology, #9718), DPP‐4 (Thermo Fisher, MA2607), collagen type II (Thermo Fisher, MA1‐37493), or MMP‐13 (R&D systems, MAB511) diluted (1:100) in 4% bovine serum albumin (1 h; 37°C). The sections were visualized using anti‐mouse secondary antibodies (Thermo Fisher, 31,437) by staining the target proteins brown (background blue‐purple color). SBP thickness was measured at five different points per rat joint and the average SBP thickness of each rat was recorded. Cartilage destruction was scored using the OA Research Society International (OARSI) grading system. Collagen type II and MMP13 were quantified using a scoring system. (See Data S1).

### 4.15 Statistical analysis

All results were reported as means and standard deviations. T‐test was conducted to assess differences in mean values between the two groups. For the comparison of more than two groups, Analysis of Variance (ANOVA) was used to determine the significance of the differences in mean between the different groups. Linear regression was conducted to assess relationship between two variables. All statistical analyses and graphs were conducted and produced using GraphPad Prism version 10 software for Windows (GraphPad Software, Boston, MA). Significance was set at *p* < 0.05.

## AUTHOR CONTRIBUTIONS

**Du Hyun Ro**: conceptualization, methodology, writing, formal analysis, investigation and revision of manuscript, **Gun Hee Cho**: conceptualization, methodology, writing, formal analysis, investigation and revision of manuscript, **Ji Yoon Kim**: formal analysis, investigation, **Seong Ki Min**: formal analysis, investigation, **Ha Ru Yang**: formal analysis, investigation, **Hee Jung Park**: formal analysis, investigation, **Sun Young Wang**: formal analysis, investigation, **You Jung Kim**: formal analysis, investigation, **Myung Chul Lee**: methodology, formal analysis, investigation, **Hyun Cheol Bae**: conceptualization, methodology, writing and revision of manuscript, **Hyuk‐Soo Han**: conceptualization, methodology, writing and revision of manuscript. All authors read and approved the final manuscript.

## FUNDING INFORMATION

This work was supported by the New Faculty Startup Fund from Seoul National University (800–2021‐0559) and the National Research Foundation of Korea (NRF) grant funded by the Korea Ministry of Science and ICT (MSIT) (NRF‐2019R1A2C1009434).

## CONFLICT OF INTEREST STATEMENT

The authors have no relevant financial or non‐financial interests to disclose.

## Supporting information


Data S1.

## Data Availability

The data that support the findings of this study are available from the corresponding author upon reasonable request.
