# Supplementary material for: Selective targeting of dipeptidyl‐peptidase 4 (DPP‐4) positive senescent chondrocyte ameliorates osteoarthritis progression
Source: Aging Cell. 2024 Mar 31;23(7):e14161. doi: 10.1111/acel.14161 (PMC11258469; doi:10.1111/acel.14161)
Supplement: Supplementary file 1 — Data S1. [file ACEL-23-e14161-s001.pdf]

Supplementary Figures

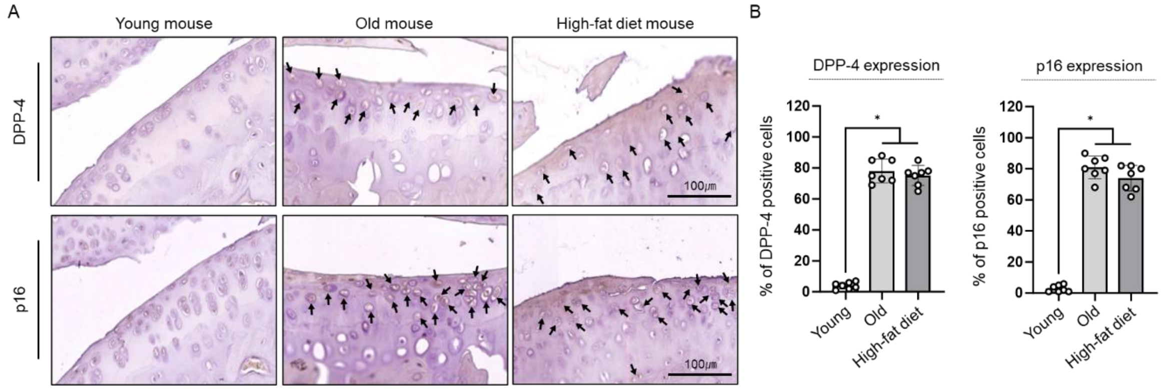

Supplementary Figure 1. DPP-4 and senescence marker expressions were upregulated in the cartilage of old mice and high-fat diet mice. (A) Representative histological analysis using IHC for DPP-4 and p16 staining to evaluate senescence chondrocyte accumulation in the cartilage of old mice and high-fat diet mice and (B) Quantification of DPP-4 and p16 positive chondrocytes (n = 7). *p < 0.05.

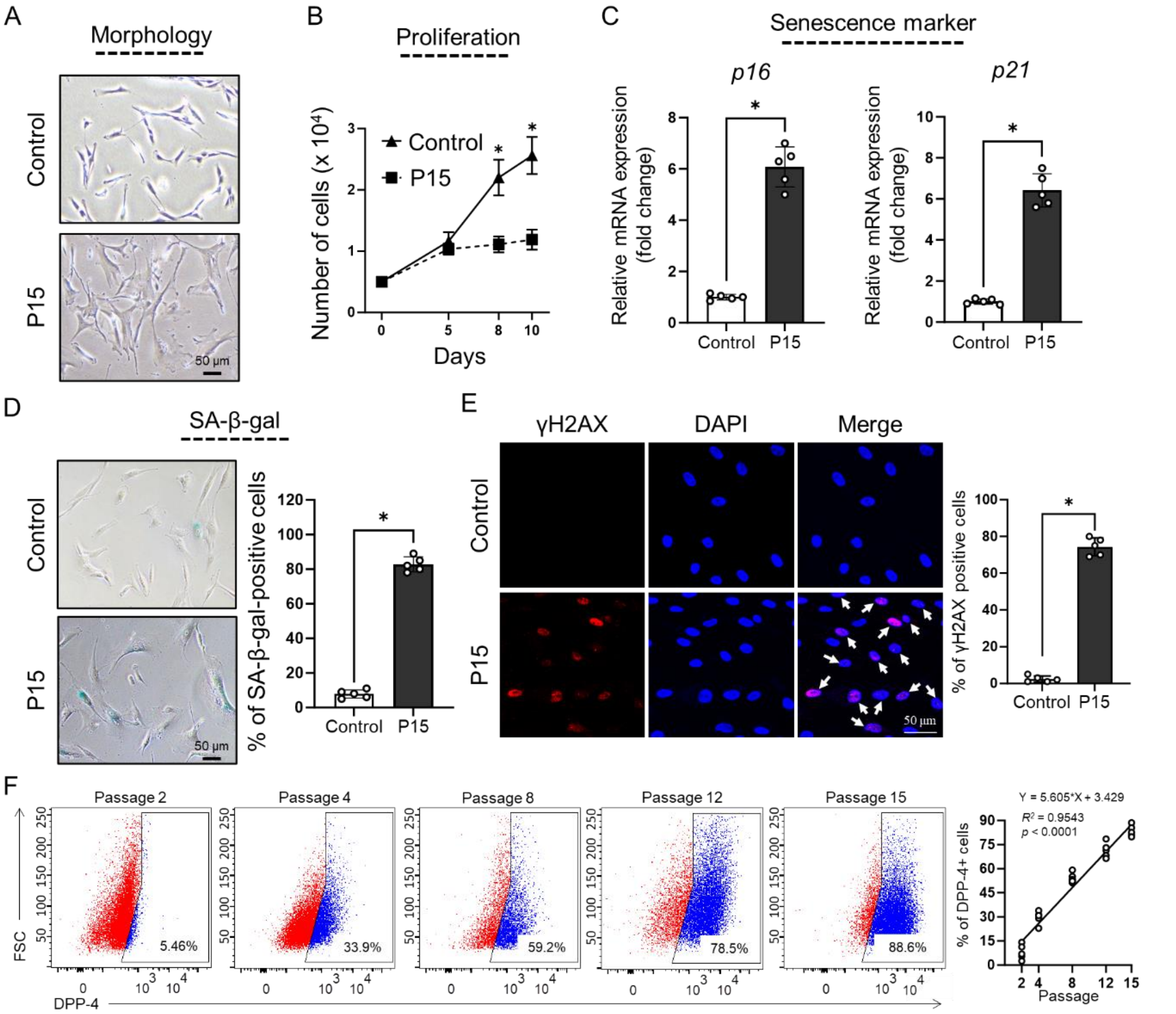

Supplementary Figure 2. Establishment of replicative senescent chondrocytes. (A) Representative morphology of replicative (passage 15; P15) senescent chondrocytes. (B) Cell growth analysis of P15 chondrocytes (n = 5). (C) The mRNA expression of p16 and p21 for P15 chondrocytes (n = 5). (D) (left) Representative SA-β-gal staining for P15 chondrocytes and (right) quantification of SA-β-gal-positive chondrocytes (n = 5). (E) (left) Representative γH2AX immunofluorescence staining for P15 chondrocytes and (right) quantification of γH2AX-positive chondrocytes (n = 5). (F) (left) Representative flow cytometry analysis of DPP-4 expression at serial passage from passage 2 to 15 and (right) the percentage of DPP-4+ chondrocytes determined by flow cytometry at each passage and linear regression analysis curves between passage and DPP-4+ chondrocytes (n = 5, respectively). *$p < 0.05$.

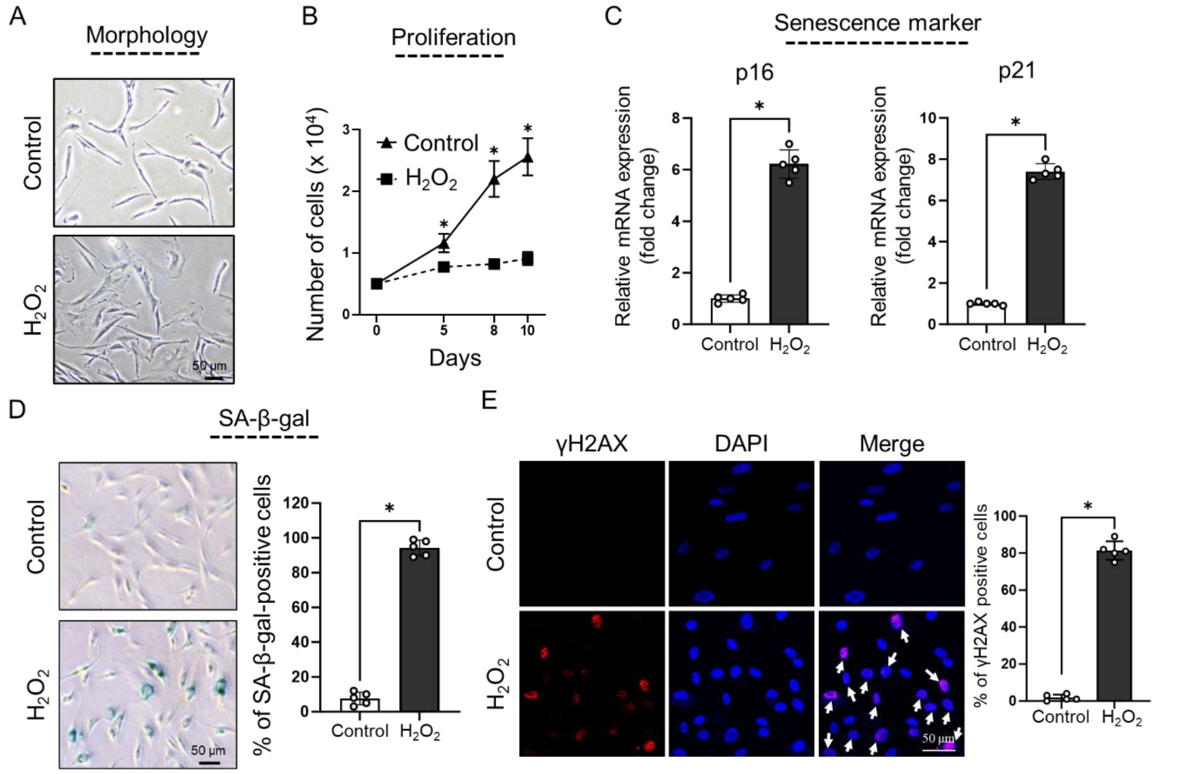

Supplementary Figure 3. Establishment of oxidative stress (H_2O_2)-induced senescent chondrocytes. (A) Representative morphology of H_2O_2-induced senescent chondrocytes. (B) Cell growth analysis of H_2O_2-induced senescent chondrocytes (n = 5). (C) The mRNA expression of p16 and p21 for H_2O_2-induced senescent chondrocytes (n = 5). (D) (left) Representative SA-β-gal staining for H_2O_2-induced senescent chondrocytes and (right) quantification of SA-β-gal-positive chondrocytes (n = 5). (E) (left) Representative γH2AX immunofluorescence staining for H_2O_2-induced senescent chondrocytes and (right) quantification of γH2AX-positive chondrocytes (n = 5). *$p < 0.05$.

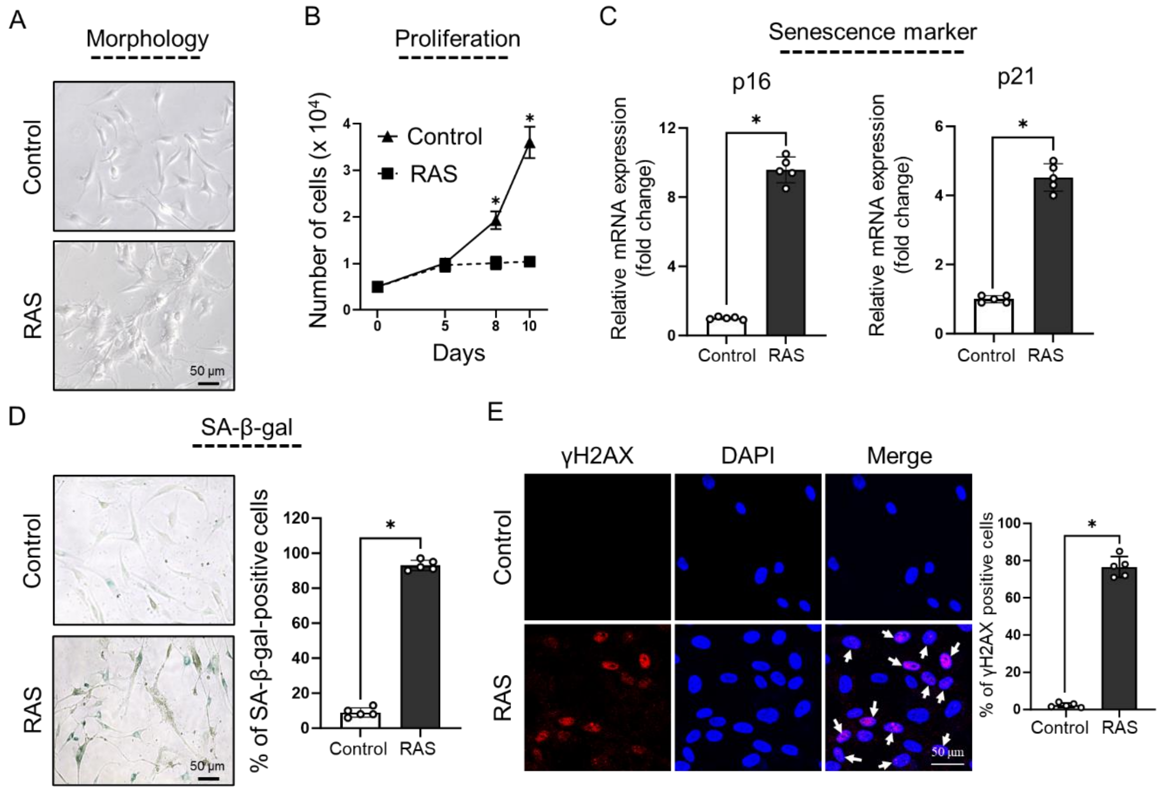

Supplementary Figure 4. Establishment of oncogene (RAS)-induced senescent chondrocytes. (A) Representative morphology of RAS-induced senescent chondrocytes. (B) Cell growth analysis of RAS-induced senescent chondrocytes (n = 5). (C) The mRNA expression of p16 and p21 for RAS-induced senescent chondrocytes (n = 5). (D) (left) Representative SA-β-gal staining for RAS-induced senescent chondrocytes and (right) quantification of SA-β-gal-positive chondrocytes (n = 5). (E) (left) Representative γH2AX immunofluorescence staining for RAS-induced senescent chondrocytes and (right) quantification of γH2AX-positive chondrocytes (n = 5). *$p < 0.05$.

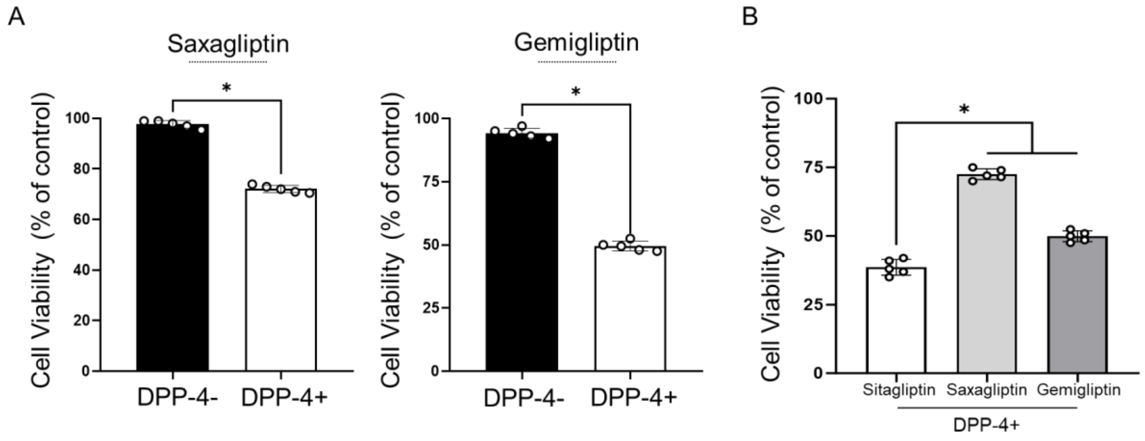

Supplementary Figure 5. Other gliptin families (saxagliptin and gemigliptin) showed senolytic effects on DPP-4 positive chondrocytes. (A) Evaluation of cell viability of DPP-4- and DPP-4+ chondrocytes upon treatment of 5 μM saxagliptin and gemigliptin (n = 5). (B) comparison of senolytic effects on DPP-4+ chondrocytes between gliptin families (sitagliptin, saxagliptin and gemigliptin) (n = 5). *$p < 0.05$.

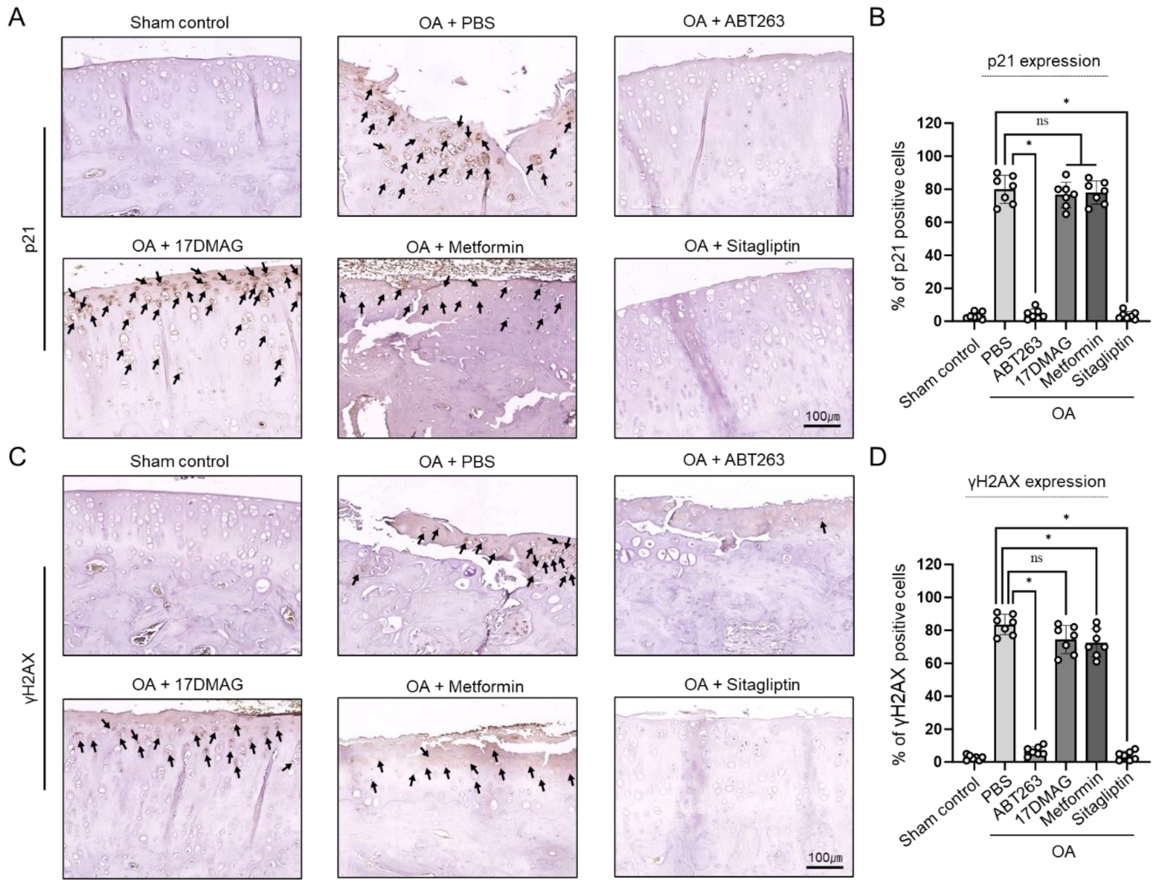

Supplementary Figure 6. Sitagliptin could specifically and effectively eliminate senescent chondrocytes. (A and C) Representative histological analysis using IHC for p21 and γH2AX staining to evaluate *in vivo* senolytic effects of ABT263, 17DMAG, metformin and sitagliptin and (B and D) Quantification of p21 and γH2AX positive cells (n = 7). *$p < 0.05$.

SUPPLEMENTARY TABLE

Table S1. TaqMan probes used for RT-qPCR.

Gene	Assay ID
COL2A1	Hs00156568_m1
COL10A1	Hs00166657_m1
ACAN	Hs00202971_m1
SOX9	Hs00165814_m1
MMP13	Hs00233992_m1
RUNX2	Hs00231692_m1
p16	Hs00923894_m1
p21	Hs00355782_m1
IL-6	Hs00174131_m1
IL-8	Hs99999034_m1
CCL2	Hs00234140_m1
BMP2	Hs00154192_m1
IGFBP7	Hs00266026_m1
TGF-β	Hs00998133_m1
GDF15	Hs00171132_m1
GAPDH	Hs02758991_g1

SUPPLEMENTARY MATERIALS & METHODS

Cell Isolation and culture from human osteoarthritic joints

Human osteoarthritic cartilage from 10 patients undergoing total knee arthroplasty and non-osteoarthritic cartilage from 10 patients who underwent tumor removal were obtained. This study was approved by our Institutional Review Board (No. H-1907-028-1045, No. 0902-021-271) and the patients provided informed consent for the donation of tissues for research purposes. Tissue was finely minced and digested with 0.025% collagenase (Sigma-Aldrich, C9891) in high-glucose Dulbecco's modified Eagle medium (DMEM; Gibco, 11965092) supplemented with 1% antibiotic- antimycotic (Gibco, 15240062) and incubated on an orbital shaker overnight at 37 °C with 5% CO_2. The digested material was filtered through a 70 µm strainer. Cells were seeded at a density of 1×10^6 in 150 mm dishes, and the medium was changed after three days. The cells were cultured in high-glucose DMEM containing 10% fetal bovine serum (FBS, Gibco, 16000044) and 1% antibiotic-antimycotic solution at 37 °C with 5% CO_2. Chondrocytes underwent passaging upon reaching approximately 80% confluence. Non-OA chondrocytes used in all the in vitro experiments were passage 2.

Chondrogenesis using pellet culture

5×10^5 cells of non-OA, DPP-4- and DPP-4+ chondrocytes (n = 5, respectively) were centrifuged at 1,500 rpm for 5 min to obtain cell pellets. Cell pellets were cultured in chondrogenic medium (LG-DMEM; Thermo Fisher, 11885084) containing 0.1 mmol/L ascorbic acid 2-phosphate, 100 nmol dexamethasone, 40 g/mL proline, 100 U/mL penicillin, 100 g/mL streptomycin, and ITS Premix (BD Biosciences, 354352) supplemented with 10 ng/ml transforming growth factor beta 1 (TGF-ß1; R&D systems, 204-B-010). The Chondrocyte pellets were undergone chondrogenesis up to 21 days. The medium was refreshed

every 3-4 days. After 21 days, pellets were harvested for histological analysis using hematoxylin and eosin (H&E) and safranin-o staining.

Establishment of replicative senescent chondrocytes

Non-OA chondrocytes (passage 2) were seeded at a density of 1×10^6 cells/150 mm dish in high-glucose DMEM containing 10% FBS and 1% antibiotic-antimycotic solution at 37 °C with 5% CO_2. When chondrocytes had proliferated to confluence of approximately 80%, chondrocytes were trypsinized, counted and seeded again at 1×10^6 cells/150 mm dish. This process was repeated 12 times until reaching passage 15. At passage 15, replicative-induced senescence chondrocytes were subjected to RT-qPCR and SA-β-gal analysis.

Establishment of oxidative stress-induced senescent chondrocytes

Non-OA Chondrocytes were plated in a 6-well plate at a density of 5×10^4 cells/well. Chondrocytes were pretreated with serum-free medium for 1h followed by treatment with 200 μM H_2O_2 for 16h (O/N). H_2O_2 containing medium was removed after 16 h and incubation with fresh medium containing 10% FBS for 24 h. This process was repeated four times every three days to produce senescent chondrocytes. After establishment of H_2O_2-induced senescent chondrocytes, RT-qPCR and SA-β-gal were conducted.

Establishment of RAS-induced senescent chondrocytes

293FT cells were seeded in 6-well plates at a density of 1×10^6 cells/well and incubated for 16 h at 37 °C. Lentiviral vectors were produced by co-transfection of 4.3 μg pLenti CMV RasV12 expression vector (Addgene plasmid # 22259), 6 μg gag-pol expression plasmid psPAX2 (Addgene plasmid # 12260), and 3 μg VSV-G envelope expression plasmid pMD2.G (Addgene plasmid # 12259). The supernatant containing assembled viral particles was collected at approximately 52 h post-transfection. Non-OA chondrocytes were seeded in

6-wells culture plate at 5×10^4 cells/well. The medium was changed with 1.0 ml of fresh complete medium and 0.5ml of the respective viral preparation was added. The cationic polymer hexadimethrine bromide (polybrene) was added to each flask to reach a final concentration of 10 μg/ml. For selection of stable chondrocytes expressing RAS, the culture medium was changed with fresh medium containing 150 μg/ml G418 (Gibco, 10131035). After finishing establishment of RAS-induced senescent chondrocytes, these chondrocytes were subjected to RT-qPCR and SA-β-gal analysis.

Cell surface marker screening by flow cytometry using BD Lyoplate

BD Lyoplate human cell surface marker screening panel (BD Biosciences, 560747) was used to screen senescent chondrocyte-specific markers. The kit contained 242 purified monoclonal antibodies against cell surface markers. The assay was performed on three donors per group according to the manufacturer's instructions. Briefly, 5×10^5 non-OA, OA chondrocytes and oxidative stress-induced senescent chondrocytes were suspended in Pharmingen Stain buffer and stained with 10 μL of specific primary antibodies for 30 min on ice. Thereafter, the cells were washed twice with Pharmingen washing buffer and stained with 100 μl of Alexa Fluor 647-labeled anti-mouse or anti- rat secondary antibody (diluted 1: 500) for 30 min. After washing, the cells were transferred to BD FACS tubes. Fluorescence was measured with a BD FACSCanto II cytometer on 10,000 cells using the FACSDiva software.

Cell sorting and flow cytometric analysis

Cell sorting was performed using anti-DPP-4 antibodies to separate DPP-4+ and DPP-4- cell populations from OA chondrocytes. Cartilage tissue from OA patients was finely minced and digested with 0.025% collagenase (Sigma-Aldrich, C9891) in high-glucose DMEM (Welgene, LM001-05) with 1% antibiotic-antimycotic solution (Gibco, 15240062) and

incubated on an orbital shaker overnight at 37 °C with 5% CO_2. The digested OA chondrocytes were filtered through a 70-μm strainer. Then, the OA chondrocytes were labeled with DPP-4-PE (BD Biosciences, 555437) or mIgG-PE (BioLegend, 405307) for 30 min at 4 °C in the dark. For the flow cytometric sorting of DPP-4+ or DPP-4- chondrocytes, anti-DPP-4-PE antibody labeled OA chondrocytes were sorted according to side scattering and PE intensity. Cell sorting and FACS analysis were performed on a Canto II flow cytometer (BD Biosciences) using FlowJo software (FlowJo version 10.2).

Cell viability assay

5×10^5 DPP-4- and DPP-4+ chondrocytes were seeded on 6-well plates. Senolytics (20 μM ABT263, 100 nM 17DMAG and 20 μM metformin) and 5 μM gliptin families (sitagliptin, saxagliptin and gemigliptin) were treated with indicated concentration for three days. The concentration of drugs used in cell viability assay was set based on previous studies using ABT263 [1], 17DMAG [2], metformin [3] and gliptin families [4]. The cell viability assay was carried out by counting the number of cells using trypan blue staining. The control groups for DPP-4- and DPP-4+ were DPP-4- and DPP-4+ chondrocytes without senolytic treatment, respectively. Data were presented as percentage of control.

Senescence-associated β-galactosidase (SA-β-gal) staining

SA-β-gal staining was conducted using SA-β-gal staining kit (Cell Signaling Technology, 9860). The culture medium was removed from 6-well plates, after which the cells were washed once with 1mL of 1× phosphate-buffered saline (PBS) and fixed with 0.5 mL of fixative solution for 10 – 15 min at room temperature. While the cells were in the fixative solution, the staining solution mixture (staining solution, staining supplement, and 20 mg/mL of X-gal in dimethyl sulfoxide) was prepared. The cells were washed twice with 1mL of 1×

PBS and incubated overnight with the staining mixture at 37 °C. The cells were observed under an inverted microscope.

Real-time quantitative polymerase chain reaction (RT-qPCR) analysis

After the extraction of total RNA using a RNeasy mini kit (Qiagen, 74104), reverse transcription was performed using the RNA to cDNA kit (TaKaRa, 639543). Real-time qPCR was conducted using TaqMan gene expression assay kits (Applied Biosystems, 4331182) in an Applied Biosystems 7500 Fast System. The TaqMan probes were purchased from Thermo Fisher, and the probes used are listed in **Table S1**. Gene expression was measured from the CT value of each gene subtracting the reference gene (GAPDH) expression ($CT_{target\ gene} - CT_{GAPDH} = \Delta CT$). The relative gene expression was determined by subtracting the $\Delta CT_{control}$ ($\Delta CT_{sample} - \Delta CT_{control} = \Delta\Delta CT$), which was used to calculate the relative expression ratio ($2^{-\Delta\Delta CT}$).

Western blot analysis

Chondrocytes were lysed with RIPA buffer (Thermo Fisher, 89900), and protein concentration was measured using a BCA assay kit (Pierce, 23227). Proteins were separated using sodium dodecyl sulfate-polyacrylamide gel electrophoresis (SDS-PAGE) and transferred to PVDF membranes. The membranes were blocked with 5% skim milk in TBST for 1 h at room temperature and probed with primary antibodies against p16 (Cell Signaling Technology, 80772S), p21 (Cell Signaling Technology, #2947), and β-actin (Sigma-Aldrich, A5441) overnight. Proteins were visualized using secondary antibodies conjugated to horseradish peroxidase (Invitrogen, 31460 [rabbit], 31437[mouse]).

Immunofluorescence staining

For immunofluorescence staining, 2×10^4 chondrocytes were seeded in Lab-Tek II 4-well chamber slide (Thermo Fisher Scientific, 154526). Chondrocytes were fixed using BD

Cytofix/Cytoperm (BD bioscience, 554722) for 30 minutes at 4 °C, washed twice with PBS, blocked using 3 % BSA in PBS for 30 minutes and incubated with primary antibody γH2AX (Cell signaling Technology, #9718) diluted (1:200) in PBS overnight at 4 °C. The slides were washed three times with PBS and then incubated with the secondary antibody (diluted 1:2000 in PBS) goat anti-rabbit Alexa Fluor 633 (Thermo Fisher Scientific, A21070) for 1 hour at 37 °C. After removing the secondary antibody and washing three times with PBS, the nucleus of chondrocytes was stained and mounted using Antifade Mounting Medium with DAPI (Vector Laboratories, H-1200) for 30 minutes. The sections were visualized using confocal microscope (Leica STELLARIS 8).

Old mice, high-fat diet mice, and rat surgical osteoarthritis induction

All experimental procedures related to the animal models were approved by the Institutional Animal Care and Use Committee (IACUC, Rat: No. 22-0038, Mouse: No. 22-0130-S1A1). All rats and mice were housed under specific pathogen-free (SPF) conditions with 1-2 animals per cage with free access to food and water and acclimatized for a week before starting experiments. Health/immune status of animals used was checked through SPF facility monitoring data. The sample size of animal experiments in this study was decided based on previously reported studies which utilized old mice, high-fat diet mice and senolytics [5, 6, 7, 8, 9]. No animals were excluded from the analysis.

For old mice, 68-week-old mice were purchased from Laboratory Animal Resource and Research Center of Korea Research Institute of Bioscience and Biotechnology (LARRC-KRIBB, South Korea). For high-fat diet mice, 24-week-old diet-induced-obesity mice were purchased from Central Lab. Animal Inc. (South Korea). Both types of mice were used without any modification or additional treatment and sacrificed after finishing acclimatization. Harvested knee were subjected to immunohistochemistry staining of DPP-4 and p16. Young

mice were used as a control group.

Destabilization of the medial meniscus (DMM) was performed in male Wistar rats (4 months; n = 7 per group, total 6 groups including sham control) to induce OA. Control and DMM surgery groups were randomly allocated. A sham surgery (control group) was conducted. All the surgeries were performed on the same day. The rats were anesthetized before surgery and maintained under 2% isoflurane. An incision was made over the medial patella to establish the DMM rat model of OA. The medial meniscus was transected after exposure of the joint capsule. The knee joint was rinsed with saline before the incision was closed using Vicryl sutures (Ethicon, D7585). Seven days after DMM surgery, treatment groups including PBS (80 μl vehicle), ABT263, 17DMAG, metformin, and sitagliptin (5 mM/80 μl, each) were randomly allocated. The concentration of injected drugs was determined based on the previous studies [5]. Each cage was labeled with surgery date and treatment date to avoid confusion. For the injection groups, intra-articular injections were administered once every three days for seven weeks. Eight weeks after surgery, the rats were subjected to motility test using treadmill to access physical performance after treatment and were then euthanized by CO_2 inhalation, and the knee joints were collected for histological analysis including OARSI score, subchondral bone plate thickness, DPP-4, p16, p21, Collagen type II, MMP13 expression.

Motility test using treadmill

Rats were accustomed to running on a treadmill for 2 days prior to be recorded. In each session, rats run for 5 minutes, and the treadmill speed was increased from 5 m/min to 25 m/min. Eight weeks after surgery before euthanized, rats run at 25 m/min until refused to run. Rats were considered exhausted when they stopped running, and the exhaustion time was recorded, and the total distance was calculated. The observer was blinded to treatment of the rats.

Histology and immunohistochemistry

Human OA (severe-damaged and mild-damaged sites) and non-OA joint tissue (n = 10, respectively) and rat and mouse knee joints (n = 7 per group) were fixed in 4% paraformaldehyde for 16 hours, dehydrated with graded concentrations of ethanol solutions, and embedded in paraffin. Sections (5 mm) were stained with Safranin-O/Fast Green. For the immunostaining experiments, paraffin- embedded sections were deparaffinized with xylene and dehydrated. The sections were then reacted with 3% H_2O_2, processed with hyaluronidase, and incubated with 10% FBS to block non- specific binding. Then, the sections were incubated with primary antibody p16 (Abcam, ab54210), p21 (ABClonal, A19094), γH2AX (Cell signaling Technology, #9718), DPP-4 (Thermo Fisher, MA2607), collagen type II (Thermo Fisher, MA1-37493), or MMP-13 (R&D systems, MAB511) diluted (1:100) in 4% bovine serum albumin (BSA) for one hour at 37 °C. The sections were visualized using anti-mouse secondary antibodies (Thermo Fisher, 31437) by brown staining for target proteins compared to background blue-purple color. Subchondral bone plate (SBP) thickness was measured at 5 different points per rat joint, and average of the SBP thickness of each rat was recorded. Cartilage destruction was scored using the OA Research Society International (OARSI) grading system. Quantification of the collagen type II and MMP13 expression was conducted using scoring system and ImageJ (version 1.53t, National Institutes of Health). The staining intensity was scored, as follows: 0 (negative), 1 (weakly positive), 2 (mildly positive), and 3 (intensely positive). The percent of positive area in cartilage was scored as follows: 0 (< 5%), 1 (5% to 25%), 2 (25% - 50%), 3 (50% - 75%), and 4 (>75%). The final expression score was determined by multiplying the positive area score to the intensity score (ranged 0 to 12). All the scoring was conducted by blinded observers using the same protocols.

Supplementary Materials & Methods Reference

1. Miura, Y., Endo, K., Komori, K., & Sekiya, I. (2022). Clearance of senescent cells with ABT-263 improves biological functions of synovial mesenchymal stem cells from osteoarthritis patients. *Stem cell research & therapy*, 13(1), 222.

2. Bertram, K. L., Narendran, N., Tailor, P., Jablonski, C., Leonard, C., Irvine, E., Hess, R., Masson, A. O., Abubacker, S., Rinker, K., Biernaskie, J., Yates, R. M., Salo, P., Narendran, A., & Krawetz, R. J. (2018). 17-DMAG regulates p21 expression to induce chondrogenesis *in vitro* and *in vivo*. *Disease models & mechanisms*, 11(10), dmm033662.

3. Chen, D., Xia, D., Pan, Z., Xu, D., Zhou, Y., Wu, Y., Cai, N., Tang, Q., Wang, C., Yan, M., Zhang, J. J., Zhou, K., Wang, Q., Feng, Y., Wang, X., Xu, H., Zhang, X., & Tian, N. (2016). Metformin protects against apoptosis and senescence in nucleus pulposus cells and ameliorates disc degeneration *in vivo*. *Cell death & disease*, 7(10), e2441.

4. Bi, J., Cai, W., Ma, T., Deng, A., Ma, P., Han, Y., Lou, C., & Wu, L. (2019). Protective effect of vildagliptin on TNF-α-induced chondrocyte senescence. *IUBMB life*, 71(7), 978–985. https://doi.org/10.1002/iub.2049

5. Jeon, O. H., Kim, C., Laberge, R. M., Demaria, M., Rathod, S., Vasserot, A. P., Chung, J. W., Kim, D. H., Poon, Y., David, N., Baker, D. J., van Deursen, J. M., Campisi, J., & Elisseeff, J. H. (2017). Local clearance of senescent cells attenuates the development of post-traumatic osteoarthritis and creates a pro-regenerative environment. *Nature medicine*, 23(6), 775–781.

6. Yang, H., Chen, C., Chen, H., Duan, X., Li, J., Zhou, Y., Zeng, W., & Yang, L. (2020). Navitoclax (ABT263) reduces inflammation and promotes chondrogenic phenotype by clearing senescent osteoarthritic chondrocytes in osteoarthritis. *Aging*, 12(13), 12750–12770.

7. Faust, H. J., Zhang, H., Han, J., Wolf, M. T., Jeon, O. H., Sadtler, K., Peña, A. N., Chung, L., Maestas, D. R., Jr, Tam, A. J., Pardoll, D. M., Campisi, J., Housseau, F., Zhou, D., Bingham, C. O., 3rd, & Elisseeff, J. H. (2020). IL-17 and immunologically induced senescence regulate response to injury in osteoarthritis. *The Journal of clinical investigation*, 130(10), 5493–5507.

8. Schafer, M. J., White, T. A., Evans, G., Tonne, J. M., Verzosa, G. C., Stout, M. B., Mazula, D. L., Palmer, A. K., Baker, D. J., Jensen, M. D., Torbenson, M. S., Miller, J. D., Ikeda, Y., Tchkonia, T., van Deursen, J. M., Kirkland, J. L., & LeBrasseur, N. K. (2016). Exercise Prevents Diet-Induced Cellular Senescence in Adipose Tissue. Diabetes, 65(6), 1606–1615.

9. Yousefzadeh, M. J., Zhao, J., Bukata, C., Wade, E. A., McGowan, S. J., Angelini, L. A., Bank, M. P., Gurkar, A. U., McGuckian, C. A., Calubag, M. F., Kato, J. I., Burd, C. E., Robbins, P. D., & Niedernhofer, L. J. (2020). Tissue specificity of senescent cell accumulation during physiologic and accelerated aging of mice. Aging Cell, 19(3).

NOTE: Please save this file locally before filling in the table, DO NOT work on the file within your internet browser as changes will not be saved. Adobe Acrobat Reader (available free here) is recommended for completion.

The ARRIVE guidelines 2.0: author checklist

The ARRIVE Essential 10

These items are the basic minimum to include in a manuscript. Without this information, readers and reviewers cannot assess the reliability of the findings.

Item		Recommendation	Section/line number, or reason for not reporting
**Study design**	1	For each experiment, provide brief details of study design including:	
		a. The groups being compared, including control groups. If no control group has been used, the rationale should be stated.	Supplementary Materials & Methods/Surgical osteoarthritis induction section
		b. The experimental unit (e.g. a single animal, litter, or cage of animals).	Supplementary Materials & Methods/Surgical osteoarthritis induction section
**Sample size**	2	a. Specify the exact number of experimental units allocated to each group, and the total number in each experiment. Also indicate the total number of animals used.	Supplementary Materials & Methods/Surgical osteoarthritis induction section
		b. Explain how the sample size was decided. Provide details of any *a priori* sample size calculation, if done.	Supplementary Materials & Methods/Surgical osteoarthritis induction section
**Inclusion and exclusion criteria**	3	a. Describe any criteria used for including and excluding animals (or experimental units) during the experiment, and data points during the analysis. Specify if these criteria were established *a priori*. If no criteria were set, state this explicitly.	Supplementary Materials & Methods/Surgical osteoarthritis induction section
		b. For each experimental group, report any animals, experimental units or data points not included in the analysis and explain why. If there were no exclusions, state so.	No exclusions
		c. For each analysis, report the exact value of *n* in each experimental group.	Supplementary Materials & Methods/Surgical osteoarthritis induction section
**Randomisation**	4	a. State whether randomisation was used to allocate experimental units to control and treatment groups. If done, provide the method used to generate the randomisation sequence.	Supplementary Materials & Methods/Surgical osteoarthritis induction section
		b. Describe the strategy used to minimise potential confounders such as the order of treatments and measurements, or animal/cage location. If confounders were not controlled, state this explicitly.	Supplementary Materials & Methods/Surgical osteoarthritis induction section
**Blinding**	5	Describe who was aware of the group allocation at the different stages of the experiment (during the allocation, the conduct of the experiment, the outcome assessment, and the data analysis).	Supplementary Materials & Methods/Surgical osteoarthritis induction section
**Outcome measures**	6	a. Clearly define all outcome measures assessed (e.g. cell death, molecular markers, or behavioural changes).	Supplementary Materials & Methods/Surgical osteoarthritis induction section
		b. For hypothesis-testing studies, specify the primary outcome measure, i.e. the outcome measure that was used to determine the sample size.	Supplementary Materials & Methods/Surgical osteoarthritis induction section
**Statistical methods**	7	a. Provide details of the statistical methods used for each analysis, including software used.	Manuscript Materials & Methods/Statistical analysis section
		b. Describe any methods used to assess whether the data met the assumptions of the statistical approach, and what was done if the assumptions were not met.	Manuscript Materials & Methods/Statistical analysis section
**Experimental animals**	8	a. Provide species-appropriate details of the animals used, including species, strain and substrain, sex, age or developmental stage, and, if relevant, weight.	Supplementary Materials & Methods/Surgical osteoarthritis induction section
		b. Provide further relevant information on the provenance of animals, health/immune status, genetic modification status, genotype, and any previous procedures.	Supplementary Materials & Methods/Surgical osteoarthritis induction section
**Experimental procedures**	9	For each experimental group, including controls, describe the procedures in enough detail to allow others to replicate them, including:	Supplementary Materials & Methods/Surgical osteoarthritis induction section, Motility test using treadmill
		a. What was done, how it was done and what was used.	Supplementary Materials & Methods/Surgical osteoarthritis induction section, Motility test using treadmill
		b. When and how often.	Supplementary Materials & Methods/Surgical osteoarthritis induction section, Motility test using treadmill
		c. Where (including detail of any acclimatisation periods).	
		d. Why (provide rationale for procedures).	Supplementary Materials & Methods/Surgical osteoarthritis induction section, Motility test using treadmill
**Results**	10	For each experiment conducted, including independent replications, report:	
		a. Summary/descriptive statistics for each experimental group, with a measure of variability where applicable (e.g. mean and SD, or median and range).	Manuscript Result
		b. If applicable, the effect size with a confidence interval.	Not applicable

49 Spadina Ave. Suite 200
Toronto ON M5V 2J1 Canada
www.biorender.com

Confirmation of Publication and Licensing Rights

February 1st, 2024
Science Suite Inc.

Subscription:	*Student Plan*
Agreement number:	*BZ26ENLKT8*
Journal name:	*Selective Targeting of Dipeptidyl-Peptidase 4 (DPP-4) Positive Senescent Chondrocyte Ameliorates Osteoarthritis Progression*

To whom this may concern,

This document is to confirm that Gunhee Cho has been granted a license to use the BioRender content, including icons, templates and other original artwork, appearing in the attached completed graphic pursuant to BioRender's Academic License Terms. This license permits BioRender content to be sublicensed for use in journal publications.

All rights and ownership of BioRender content are reserved by BioRender. All completed graphics must be accompanied by the following citation: “Created with BioRender.com”.

BioRender content included in the completed graphic is not licensed for any commercial uses beyond publication in a journal. For any commercial use of this figure, users may, if allowed, recreate it in BioRender under an Industry BioRender Plan.

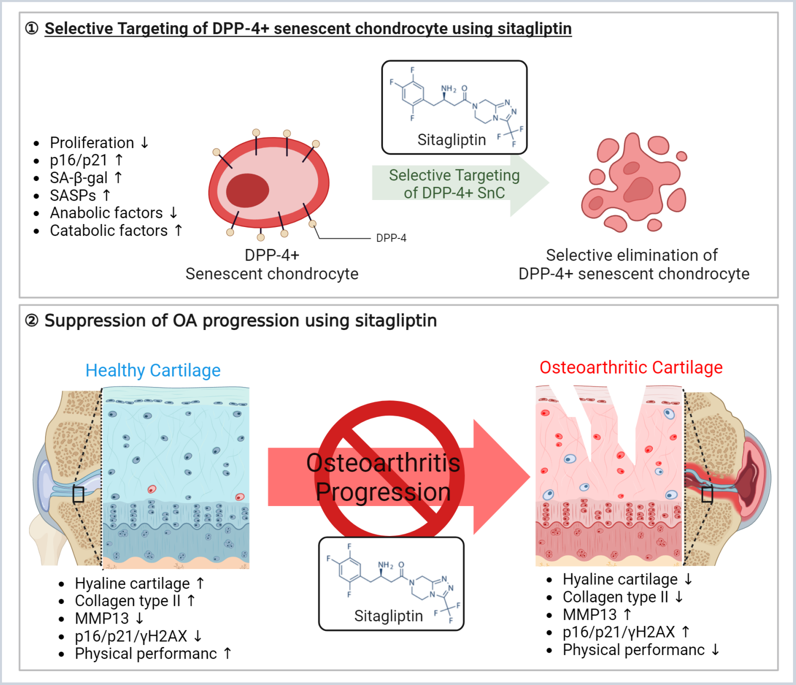

For any questions regarding this document, or other questions about publishing with BioRender refer to our BioRender Publication Guide, or contact BioRender Support at support@biorender.com.
